# A Novel GmSIN1‐GmRNF1a‐GmCSN5a Module Determines Soybean Salt Tolerance and Yield Under Saline Soil Conditions

**DOI:** 10.1002/advs.74216

**Published:** 2026-02-15

**Authors:** Jinlong Xu, Mengqi Shan, Xinxin Yang, Rongmei Zhao, Mengke Zhang, Guohua Bao, Lili Wang, Mingru Li, Wenjiao Zhi, Muzi Wang, Tianshi Liu, Xiaojian Zheng, Wenyu Zheng, Qing Lu, Shuo Li, Fengning Xiang

**Affiliations:** ^1^ The Key Laboratory of Plant Development and Environmental Adaptation Biology, Shandong Key Laboratory of Precision Molecular Crop Design and Breeding, Ministry of Education, Shandong Provincial Key Laboratory of Plant Stress Biology and Genetic Improvement; School of Life Sciences Shandong University Qingdao China

## Abstract

Soybean (*Glycine max*) is a major source of dietary protein and vegetable oil, but its production is severely reduced by salt stress. The regulatory mechanisms and utilization of salt‐tolerant genes have not been deeply studied. Salt‐Induced NAC 1 (GmSIN1) positively regulates salt tolerance in soybean, enhancing growth and yield in saline soil. Here, we found that GmSIN1 is degraded via the 26S proteasome pathway, and this process is suppressed by salt. GmRNF1a mediates the ubiquitination‐dependent degradation of GmSIN1 as an E3 ubiquitin ligase, whereas GmCSN5a (a homolog of COP9 signalosome subunit) directly inhibits its E3 ligase activity. *GmRNF1a* negatively regulates salt tolerance while *GmCSN5a* functions as a positive regulator. We further identified elite haplotypes of *GmSIN1*, *GmRNF1a*, and *GmCSN5a* that associate with grain weight per plant under both normal and saline conditions. Gene‐pyramided lines carrying elite alleles (*GmSIN1^Hap1^*‐*GmRNF1a^Hap2^*‐*GmCSN5a^Hap1^*) exhibit boosted grain yield under both conditions. In conclusion, our study reveals that the GmSIN1‐GmRNF1a‐GmCSN5a module enhances soybean salt tolerance by maintaining GmSIN1 orthostasis. Pyramiding elite haplotypes establishes an innovative haplotype‐based breeding strategy for developing salt‐tolerant and high‐yielding soybean cultivars by harnessing natural variation.

## Graphical abstract

Soybean production is severely threatened by salt stress. This study reveals that the GmSIN1‐GmRNF1a‐GmCSN5a module enhances salt tolerance by stabilizing the GmSIN1 protein. GmRNF1a acts as an E3 ligase to ubiquitinate GmSIN1 for degradation, a process inhibited by GmCSN5a. Pyramiding elite haplotypes of these genes significantly boosts yield under both normal and saline conditions, offering a novel haplotype‐based breeding strategy for salt‐tolerant, high‐yielding soybeans.

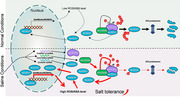

## 1 Introduction

Soil salinity is a major abiotic stress that significantly reduces global crop production [1]. Salt stress triggers ionic, osmotic, and secondary stresses, leading to cellular damage in plants [2, 3, 4]. Plants have evolved the ability to sense salt stress, transduce salt‐stress signals into cells, and respond to these signals [5, 6]. As plants vary widely in their salt tolerance, a major goal of crop breeding is to improve salt tolerance in elite cultivars.

Soybean (*Glycine max*), a major source of dietary protein and vegetable oil, is typically sensitive to salt [7]. To date, over 100 genes involved in salt tolerance in soybean have been characterized, including those encoding ion transporters, transcription factors (TFs), and other stress‐associated proteins [8]. Although the functions of these genes in salt tolerance are known, their genetic potential remains underutilized. Five elite salt‐tolerant haplotypes have been discovered in genes encoding cation/proton antiporters (*GmCHX1*, *GmNPF*) [9, 10], cation diffusion facilitators (*GmCDF1*) [11], and transcriptional regulators (*GmERD15B*, *GmARF16*) [7, 12]. Increasing the number of elite salt‐tolerant haplotypes and using these genetic variants in breeding programs is important for developing cultivars with improved growth in saline soil [13].

Transcription factors in the NAC family [14, 15]are important regulators of salinity stress responses in plants, with salt‐tolerance functions characterized in *Arabidopsis thaliana* [16, 17, 18], wheat (*Triticum aestivum*) [19, 20], and rice (*Oryza sativa*) [21]. Among the 226 NAC genes in soybean, a limited number have been shown to function in salt tolerance [22, 23, 24, 25, 26, 27]. Although these NAC TF genes are known to be transcriptionally regulated in response to salt stress, their regulation at the protein level through post‐translational modifications, particularly ubiquitin‐proteasome system (UPS)‐mediated proteasomal degradation, remains underexplored.

The UPS, an essential regulator of post‐translational modifications, fine‐tunes plant salinity tolerance by dynamically regulating protein stability and activity [28]. This process is executed through an evolutionarily conserved E1‐E2‐E3 enzyme cascade that directs the proteasomal degradation of substrate proteins or alters their activities [29]. Substrate specificity within this pathway is primarily determined by E3 ubiquitin ligases through the precise recognition of their targets. Plant E3 ligases are classified into four groups: U‐box, homologous to the E6‐AP carboxyl terminus (HECT), really interesting new gene (RING), and Cullin‐RING E3 ligases (CRLs) [30, 31]. These E3 ligases mediate plant responses to abiotic stress through substrate‐specific ubiquitination events [32, 33]. The roles of E3 ligases in stress responses in soybean are not well understood; however, one study reported that GmCHYR16 functions in bicarbonate tolerance through the ubiquitination‐dependent regulation of GmERF71 [34].

Dysregulated activation or substrate misidentification by E3 ligases often results in aberrant protein proteolysis [35]. Plants have evolved a mechanism that regulates E3 ligase activity and counteracts this effect [36]. The COP9 signalosome (CSN) is an evolutionarily conserved protein complex found in plants and animals comprising eight subunits (CSN1–CSN8) [37, 38]. CSN5 is the key subunit that catalyzes the de‐neddylation of the Cullin1 subunit in the Cullin‐RING E3 ligase (CRL) complex by removing the ubiquitin‐like protein NEDD8 [39]. However, the interaction mechanisms among the CSN and E3 ubiquitin ligases and their substrates remain largely unknown.

We previously determined that the NAC TF Salt‐Induced NAC1 (GmSIN1) regulates salt tolerance and yield in soybean [25]. Here, we demonstrate that the RING‐type E3 ligase GmRNF1a mediates GmSIN1 degradation via the 26S proteasome under normal conditions, while salt stress triggers GmCSN5a to inhibit GmRNF1a, thereby stabilizing GmSIN1 by blocking ubiquitination. This GmRNF1a‐GmCSN5a antagonistic ubiquitination system regulates GmSIN1 proteostasis, and pyramiding their elite haplotypes offers a promising strategy for breeding salt‐tolerant soybean cultivars.

## 2 Results

### 2.1 GmSIN1 stability Is Preserved Under Salt Stress due to Suppressed Ubiquitin‐Proteasome‐Mediated Degradation

In our previous study, we found that *GmSIN1* overexpression (OE) enhances salt tolerance and yield of soybean in saline soil, whereas *GmSIN1‐RNAi* silencing has the opposite effect [25]. To confirm the role of GmSIN1 in regulating salt tolerance, we generated double‐knockout mutants (*gmsin1 gmsin1h*) using CRISPR‐Cas9 genome editing to target both *GmSIN1* and its paralog *GmSIN1h* in the ‘Williams 82’ (W82) background. Two independent lines carried frameshift mutations that resulted in premature termination of translation: 4‐bp or 76‐bp deletions in the first exon of *GmSIN1* combined with 4‐bp or 2‐bp deletions in the first exon of *GmSIN1h* (Figure S1A–C), resulting in premature termination of translation, respectively (Figure S1D). Under normal conditions, the *gmsin1 gmsin1h* mutants exhibited significant reductions in plant height, pod number, grain number, and grain weight per plant (Figure S2A–E). Field trials conducted in 2024 and 2025 under two salinity levels (0.2–0.3 g and 0.3–0.4 g total soluble salts per 100 g of dry soil) revealed compromised plant height, pod number, and grain weight (Figure S2F–Q). In summary, *GmSIN1* plays an indispensable role in soybean salt tolerance, making it imperative to investigate its regulatory molecular mechanisms.

**FIGURE 1:**
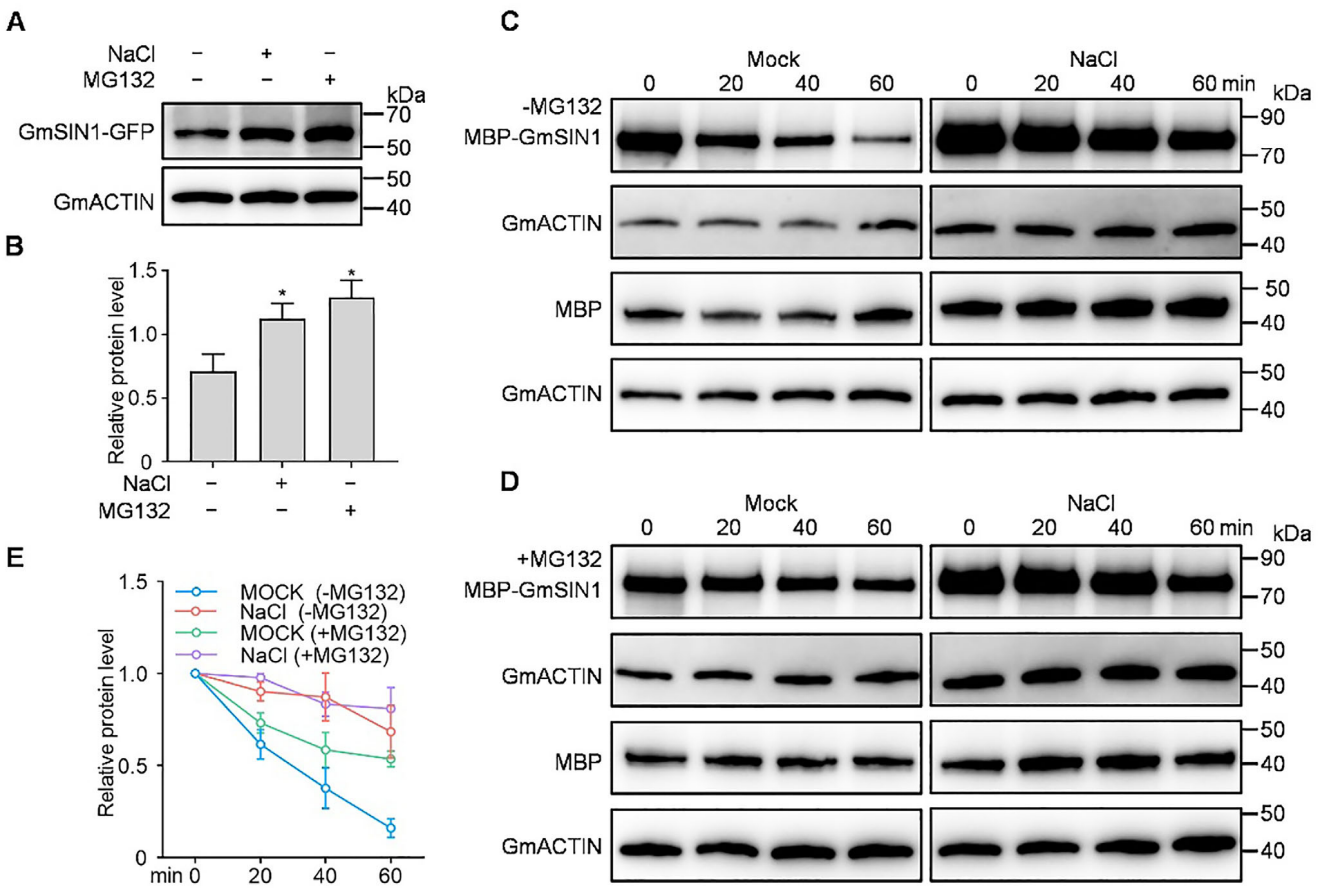
Salt stress stabilizes GmSIN1 by inhibiting its ubiquitin‐mediated 26S proteasomal degradation. (A) Immunoblot showing GmSIN1 protein accumulation in response to salt (NaCl) and proteasome inhibitor (MG132) treatments. GmACTIN served as the loading control. (B) Quantitative analysis of GmSIN1 band intensities from (A). Data are normalized to the untreated control (set to 1). (C) In vitro degradation assay of GmSIN1 using root protein extracts from wild‐type (W82) plants with or without prior NaCl treatment. (D) In vitro degradation assay showing the effect of MG132 on GmSIN1 stability, using root protein extracts as in (C). (E) Quantification of GmSIN1 band intensities from degradation assays in (C) and (D). Intensity at time 0 min was set to 1.0 for each reaction. GmACTIN was used as an internal control in (A), (C), and (D); MBP served as a negative control in (C) and (D). Data in (D) are presented as mean ± SEM (n = 4 biological replicates). These data were analyzed by one‐way ANOVA followed by Fisher's LSD test (two‐sided); asterisks indicate significant differences (^*^ *p* < 0.05). Data in e are presented as mean ± SEM (n = 3 biological replicates).

Previous study has shown that *GmSIN1* is transcriptionally upregulated in response to salt stress [25], but its post‐translational regulation remains uncharacterized. To examine GmSIN1 protein dynamics under salinity, we generated transgenic soybean hairy roots in the W82 background that harbor the *35Spro::GmSIN1‐GFP* construct. We treated the transgenic hairy roots with NaCl (150 mM, 12 h) or the proteasome inhibitor MG132 (50 µM, 12 h) and extracted total proteins for immunoblotting. We detected greater GmSIN1‐GFP stability under these treatments compared to the controls (Figure 1A–B), indicating that GmSIN1 is more stable under salt stress and might be degraded via the 26S UPS.

To confirm that GmSIN1 is degraded via the 26S proteasome, we performed cell‐free degradation assays using total protein extracts from W82 roots. MBP‐GmSIN1 exhibited time‐dependent degradation under control conditions, whereas MG132 treatment significantly attenuated this process, as revealed by immunoblotting (Figure 1C–E), confirming that GmSIN1 degradation is mediated by the 26S proteasome. To determine whether GmSIN1 undergoes ubiquitin‐proteasome‐dependent degradation under salt stress, we repeated the assays using total protein extracts from W82 roots treated with NaCl (150 mM for 12 h). GmSIN1 degradation was markedly inhibited in wild‐type W82 extracts under NaCl treatment, while combined treatment with NaCl and MG132 failed to produce a significant additive effect (Figure 1C–E). MBP, used as a control, did not show degradation under any reaction conditions (Figure 1C–E). This biochemical evidence indicates that salt stress stabilizes GmSIN1 by impairing its UPS‐mediated degradation.

### 2.2 GmSIN1 Physically Interacts with the E3 Ligase GmRNF1a and the COP9 Signalosome Subunit GmCSN5a

**FIGURE 2:**
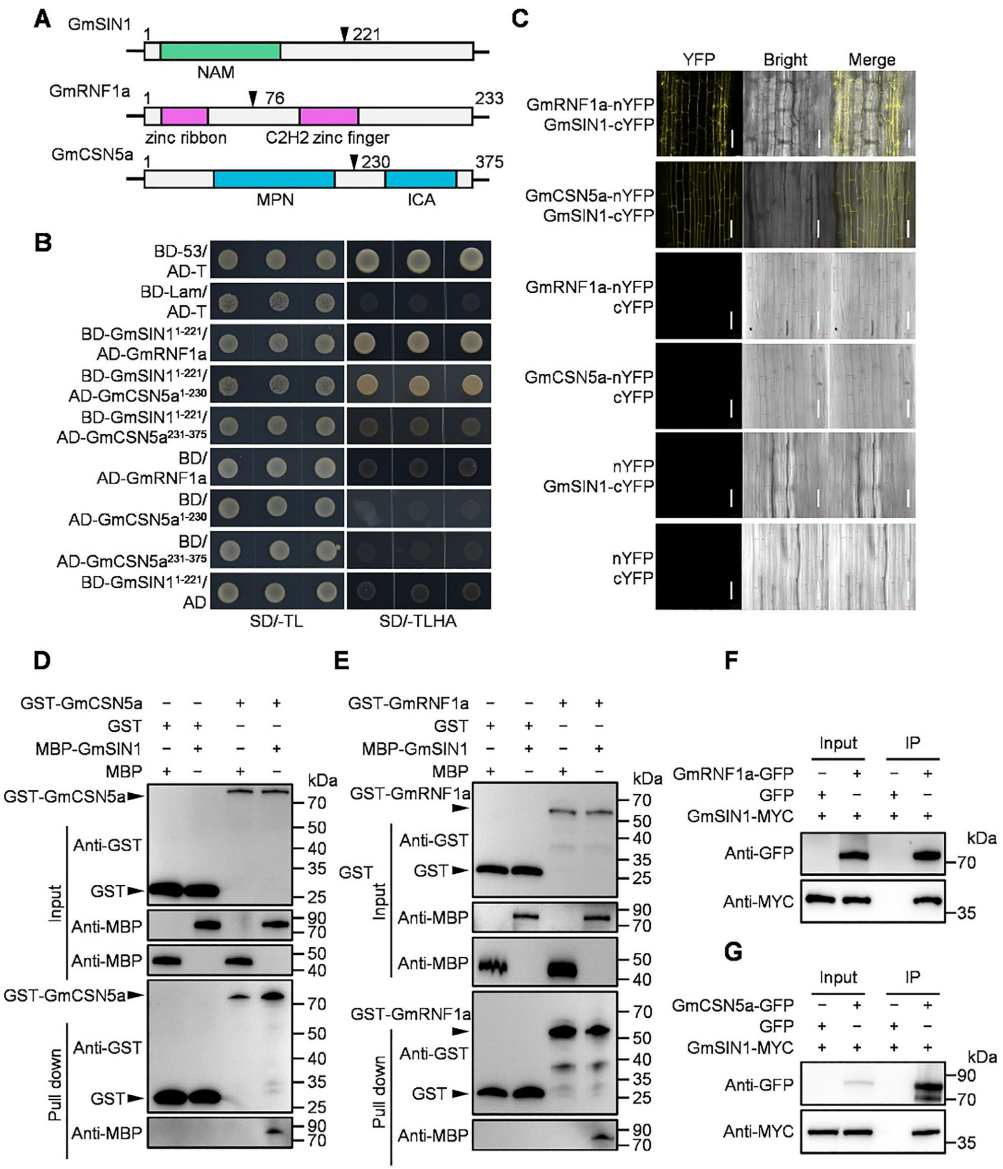
GmSIN1 interacts with GmRNF1a and GmCSN5a in vivo and in vitro. (A) Schematic representation of the protein structures and major functional domains of GmSIN1, GmRNF1a, and GmCSN5a. (B) Yeast two‐hybrid (Y2H) assays showing that GmSIN1 interacts with GmRNF1a and GmCSN5a (MPN domain). Yeast transformants were grown on synthetic defined (SD) medium lacking Leu and Trp (‐Leu/‐Trp) or lacking Ade, His, Leu, and Trp (‐Ade/‐His/‐Leu/‐Trp). BD‐53/AD‐T and BD‐Lam/AD‐T served as positive and negative controls, respectively. (C) Bimolecular fluorescence complementation (BiFC) assays confirming the interaction between GmSIN1 and GmRNF1a or GmCSN5a in *Agrobacterium rhizogenes*‐mediated transient expression in soybean hairy roots. Scale bar = 20 µm. (D, E) Pull‐down assays demonstrating direct physical interactions. Recombinant MBP‐GmSIN1 was specifically pulled down by GST‐GmRNF1a (D) or GST‐GmCSN5a (E) immobilized on glutathione resin. MBP and GST tags alone served as negative controls. (F, G) Co‐immunoprecipitation (Co‐IP) assays validating the interactions in vivo. Constructs were co‐expressed in *N. benthamiana* leaves via *Agrobacterium*‐mediated transient expression. Total proteins were extracted 72 h post‐infiltration and immunoprecipitated with anti‐GFP agarose beads. Immunoblotting was performed using anti‐MBP, anti‐GST, anti‐GFP, and anti‐MYC antibodies as indicated. All experiments were repeated at least three times with consistent results. Representative images or blots are shown.

To investigate the mechanism underlying the stability of GmSIN1 under salt stress, we performed a yeast two‐hybrid (Y2H) screen using a truncated GmSIN1 variant (GmSIN1^1–221^) lacking the transcriptional activation domain (Figure 2A). We identified two interactors: GmRNF1a, a RING‐type E3 ubiquitin ligase with an N‐terminal zinc ribbon domain and a C‐terminal C2H2 zinc finger domain; and GmCSN5a, a COP9 signalosome subunit with an N‐terminal MPN domain and a C‐terminal ICA domain (Figure 2A). Domain‐specific Y2H assays revealed that GmSIN1^1–221^ (harboring the NAM domain) binds to both GmRNF1a and GmCSN5a^1–230^ (containing the MPN domain) (Figure 2B). To validate these interactions in vivo, we performed subcellular co‐localization experiments and bimolecular fluorescence complementation (BiFC) assays in transgenic soybean hairy roots. Co‐expression of *GmSIN1‐GFP* with *GmRNF1a‐RFP* or *GmCSN5a‐RFP* resulted in overlapping GFP and RFP signals (Figure S3A,B), while co‐expression of *GmSIN1‐cYFP* with *GmRNF1a‐nYFP* or *GmCSN5a‐nYFP* resulted in YFP fluorescence due to the reconstitution of YFP (Figure 2C), thereby confirming their physical interaction. This direct binding was further confirmed by in vitro GST pull‐down assays, where MBP‐GmSIN1 specifically interacted with GST‐GmRNF1a and GST‐GmCSN5a (Figure 2D,E). Co‐immunoprecipitation (Co‐IP) experiments in *Nicotiana benthamiana* leaves further validated these interactions (Figure 2F,G). These results demonstrate that GmSIN1 interacts with both GmRNF1a and GmCSN5a.

### 2.3 The GmRNF1a‐Mediated Ubiquitination and Proteasomal Degradation of GmSIN1 Are Suppressed Under Salt Stress

*GmRNF1a* encodes a RING‐type E3 ligase containing a conserved Cys‐Xn‐His‐Xn‐His‐Xn‐Cys motif (positions 123–135) (Figure S4A). To assess its enzymatic activity, we performed in vitro ubiquitination assays. GST‐GmRNF1a (in the presence of E1 [UBE1], E2 [UBCH5], and ubiquitin) catalyzed the formation of polyubiquitin chains, whereas its catalytic mutants (GST‐GmRNF1a^4Y^ harboring C127Y/H129Y/C132Y/H135Y mutations) exhibited no detectable E3 ligase activity under identical conditions (Figure S4A,B). Consistently, GmCSN5a also showed no detectable E3 ligase activity (Figure S4C).

**FIGURE 3:**
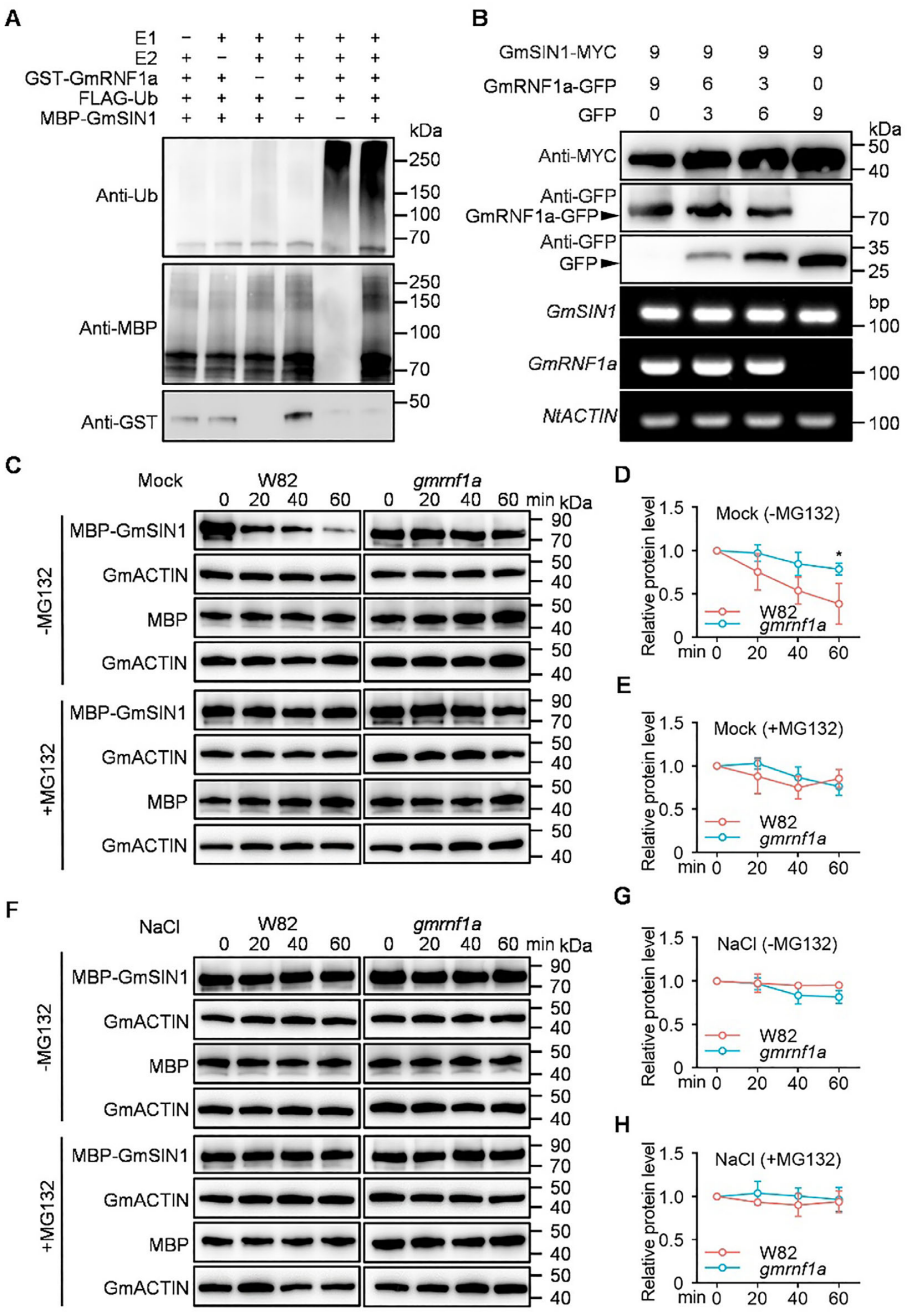
GmRNF1a ubiquitinates GmSIN1 and modulates its abundance. (A) GmRNF1a acts as an E3 ubiquitin ligase for GmSIN1 polyubiquitination in vitro, assayed in a reconstituted system containing E1, E2, Ub, GST‐RNF1a, and MBP‐GmSIN1. (B) GmRNF1a reduces GmSIN1 protein abundance in vivo. SIN1‐MYC and RNF1a‐GFP (or GFP control) were co‐expressed in *N. benthamiana* leaves at the indicated *Agrobacterium* ratios. *NtACTIN* was used as an internal control. (C) Cell‐free degradation of MBP‐GmSIN1 incubated with root proteins from W82 or *gmrnf1a‐1* plants, with or without 50 µM MG132. MBP served as a negative control. (D, E) Quantitative analysis of MBP‐GmSIN1 degradation in c. Relative protein levels are shown (0 min set to 1.0). (F) Cell‐free degradation assays using root proteins from NaCl‐treated W82 or *gmrnf1a‐1* plants. (G, H) Quantitative analysis of MBP‐GmSIN1 degradation in (F). Relative protein levels are shown (0 min set to 1.0). Anti‐ubiquitin and anti‐MBP immunoblotting detected polyubiquitination in (A); anti‐GFP and anti‐MYC immunoblotting assessed protein levels in (B). RT‐PCR confirmed transcript levels in (B). Experiments in (A) and (B) were performed three times with similar results. Data in (D), (E), (G), (H) are presented as mean ± SD (n = 3 biological replicates). Data in (D) were analyzed by *Student t*‐test (two‐side), asterisks indicate significant differences (^*^ *p* < 0.05).

In in vitro ubiquitination assays, GST‐GmRNF1a directly promoted the polyubiquitination of MBP‐GmSIN1 (Figure 3A). To investigate the impact of GmRNF1a on GmSIN1 stability, we performed transient co‐expression assays in *N. benthamiana* leaves. Co‐expression of *GmRNF1a‐GFP* and *GmSIN1‐MYC* (both driven by the 35S promoter) revealed an inverse association: GmSIN1‐MYC accumulation increased as GmRNF1a‐GFP levels decreased (Figure 3B), indicating that GmRNF1a negatively regulates GmSIN1 stability.

To investigate the direct GmRNF1a‐mediated ubiquitination and subsequent degradation of GmSIN1, we generated knockout mutants (*gmrnf1a*, T2 generation) through CRISPR‐Cas9 genome editing targeting *GmRNF1a* in the W82 background. Two independent lines carried frameshift mutations: 4‐bp or 53‐bp deletions in the first exon of *GmRNF1a*, resulting in premature termination of translation (Figure S5A–C). The *gmrnf1a* mutants did not exhibit obvious alterations in agronomic traits (e.g., plant height, pod number, grain number, or grain weight per plant) compared to the wild‐type W82 under normal conditions (Figure S5D–H). To confirm the degradation of GmSIN1 by GmRNF1a, we performed cell‐free degradation assays using total protein extracts from wild‐type W82 and *gmrnf1a‐1* roots. MBP‐GmSIN1 exhibited time‐dependent degradation when incubated with wild‐type W82 root extract; however, this degradation was significantly attenuated in reactions containing *gmrnf1a‐1* extract, as revealed by immunoblotting (Figure 3C–E).

To investigate the degradation of GmSIN1 by GmRNF1a under salt stress, we repeated the assays using total protein extracts from wild‐type W82 and *gmrnf1a‐1* roots treated with NaCl (150 mM for 12 h). GmSIN1 degradation was markedly inhibited in both wild‐type W82 and *gmrnf1a‐1* extracts under NaCl treatment, while the combined treatment with NaCl and MG132 failed to produce a significant additive effect (Figure 3F–H). MBP, used as a control, did not show degradation under any reaction conditions (Figure 3C–H). These results indicate that under normal conditions, GmRNF1a drives ubiquitin‐proteasome‐dependent GmSIN1 degradation, which is specifically inhibited under salt stress.

### 2.4 GmCSN5a Inhibits GmSIN1 Ubiquitination by Suppressing the E3 Ligase Activity of GmRNF1a

**FIGURE 4:**
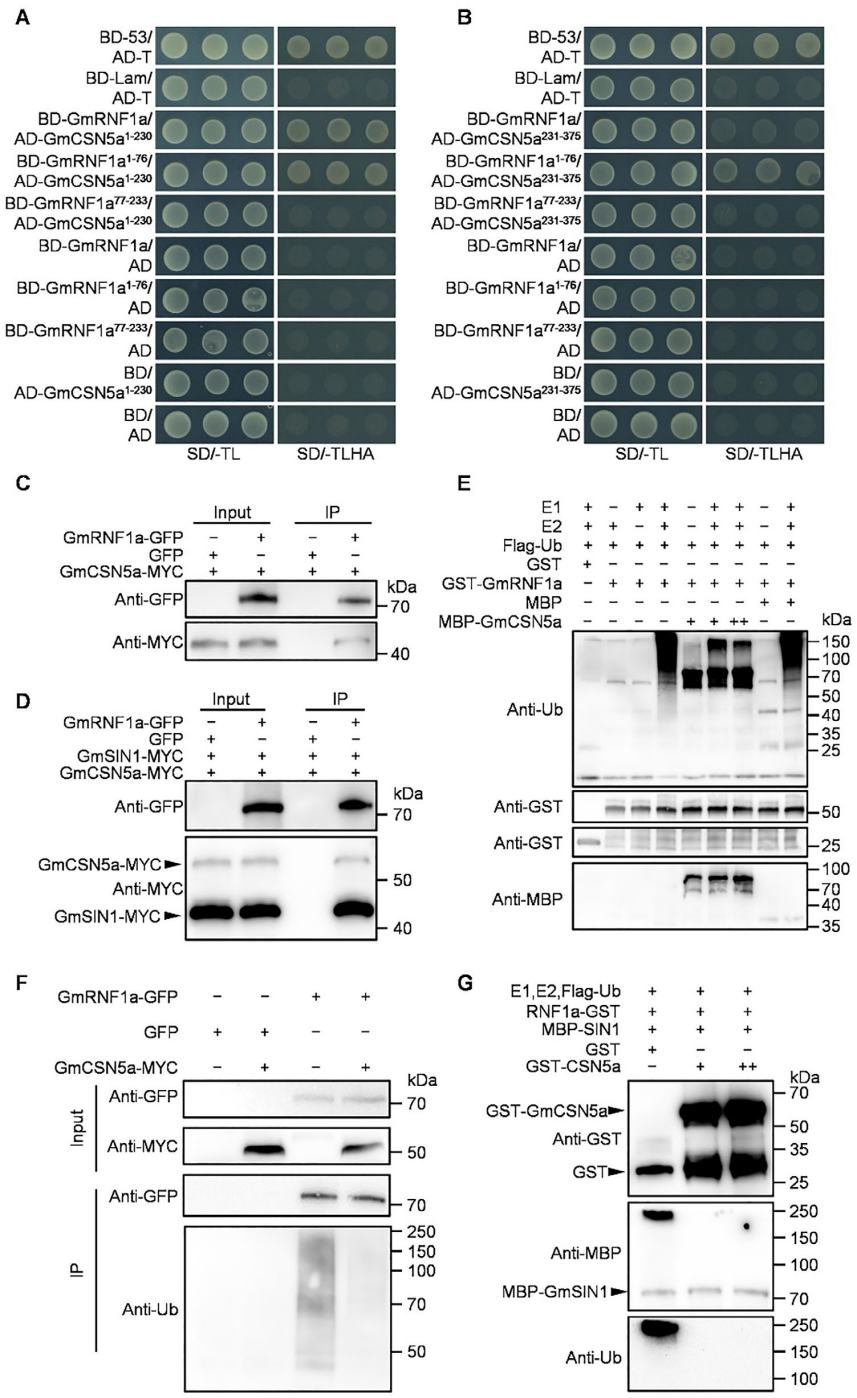
GmCSN5a inhibits GmSIN1 ubiquitination by suppressing the E3 ligase activity of GmRNF1a. (A, B) Yeast two‐hybrid (Y2H) assays showing interaction between the N‐terminal zinc ribbon domain of GmRNF1a and the MPN or ICA domains of GmCSN5a. (C) Co‐immunoprecipitation (Co‐IP) assays confirming the in vivo interaction between GmRNF1a and GmCSN5a. (D) Co‐IP assays demonstrating that GmRNF1a interacts with both GmSIN1 and GmCSN5a in vivo. (E) GmCSN5a inhibits the E3 ligase activity of GmRNF1a in vitro, assayed in a reconstituted ubiquitination system containing E1, E2, Ub, GST‐RNF1a, and MBP‐GmCSN5a. GST and MBP served as negative controls. (F) GmCSN5a inhibits the E3 ligase activity of GmRNF1a in vivo. The indicated constructs were co‐expressed in *N. benthamiana* leaves, followed by Co‐IP with anti‐GFP beads at 72 hpi. (G) GmCSN5a suppresses GmRNF1a‐mediated polyubiquitination of GmSIN1 in vitro. Immunoblotting with anti‐Ub, anti‐GST, anti‐MBP, anti‐GFP, or anti‐MYC antibodies was used as indicated.

CSN5a regulates the E3 ligase activity in CRL complexes [40, 41]. To determine whether GmCSN5a regulates GmRNF1a‐mediated ubiquitination, we first assessed the interaction between these two proteins using domain‐specific Y2H assays. GmRNF1a^1–76^ (harboring the zinc ribbon domain) interacts with both GmCSN5a^1–230^ (harboring the MPN domain) and GmCSN5a^231–375^ (harboring the ICA domain). However, GmRNF1a^77–233^ (harboring the C2H2 zinc finger domain) does not interact with either domain of GmCSN5a (Figure 4A,B). BiFC, GST pull‐down, and Co‐IP assays all demonstrated the interaction between GmCSN5a and GmRNF1a (Figure 4C; Figure S6A,B). Notably, Co‐IP assays showed that GmRNF1a simultaneously interacted with both GmSIN1 and GmCSN5a (Figure 4D). These findings align with the interactions between GmSIN1 and GmRNF1a, as well as between GmSIN1 and GmCSN5a (Figure 2B–G), suggesting that these proteins are regulated by a coordinated network.

To investigate the role of GmCSN5a in GmRNF1a‐mediated ubiquitination, we performed in vitro ubiquitination assays. In a reconstituted ubiquitination system (E1, E2, GmRNF1a/E3), recombinant MBP‐GmCSN5a suppressed the polyubiquitination of GmRNF1a in a concentration‐dependent manner, whereas the MBP control did not (Figure 4E; Figure S7A,B). To validate this finding *in planta*, we transiently co‐expressed GmCSN5a‐MYC and GmRNF1a‐GFP in tobacco *N. benthamiana* leaves, followed by immunoprecipitation using an anti‐GFP antibody. These assays demonstrated that GmCSN5a suppresses the E3 ligase activity of GmRNF1a (Figure 4F). To further demonstrate the direct inhibition of GmRNF1a‐mediated ubiquitination by GmCSN5a, we performed in vitro ubiquitination assays using purified recombinant proteins containing the key domains and truncated forms of GmCSN5a. We found that the MPN domain of GmCSN5a strongly inhibits GmRNF1a‐mediated ubiquitination, whereas deletion of this domain (ΔMPN) completely abolishes the suppression (Figure S7C). These results conclusively demonstrate that GmCSN5a directly inhibits GmRNF1a‐mediated ubiquitination through its MPN domain. Furthermore, in an in vitro E1‐E2‐GmRNF1a‐GFP ubiquitination assay, GmCSN5a markedly decreased the ubiquitination level of GmSIN1 (Figure 4G). Collectively, our data demonstrate the formation of a functional tripartite complex consisting of GmCSN5a, GmRNF1a, and GmSIN1, by which GmCSN5a inhibits GmRNF1a‐mediated ubiquitination of GmSIN1 in an MPN domain‐dependent manner.

### 2.5 *GmRNF1a* Negatively Regulates Salt Tolerance in Soybean

To investigate the expression patterns of *GmRNF1a* and *GmCSN5a*, we performed RT‐qPCR using RNA extracted from various tissues of the wild‐type W82. Both genes were ubiquitously expressed, with *GmRNF1a* showing the highest transcript levels in leaves and *GmCSN5a* in cotyledons (Figure S8A,C). Both *GmRNF1a* and *GmCSN5a* were upregulated under salt stress in soybean roots (150 mM NaCl for 12 h) (Figure S8B,D).

**FIGURE 5:**
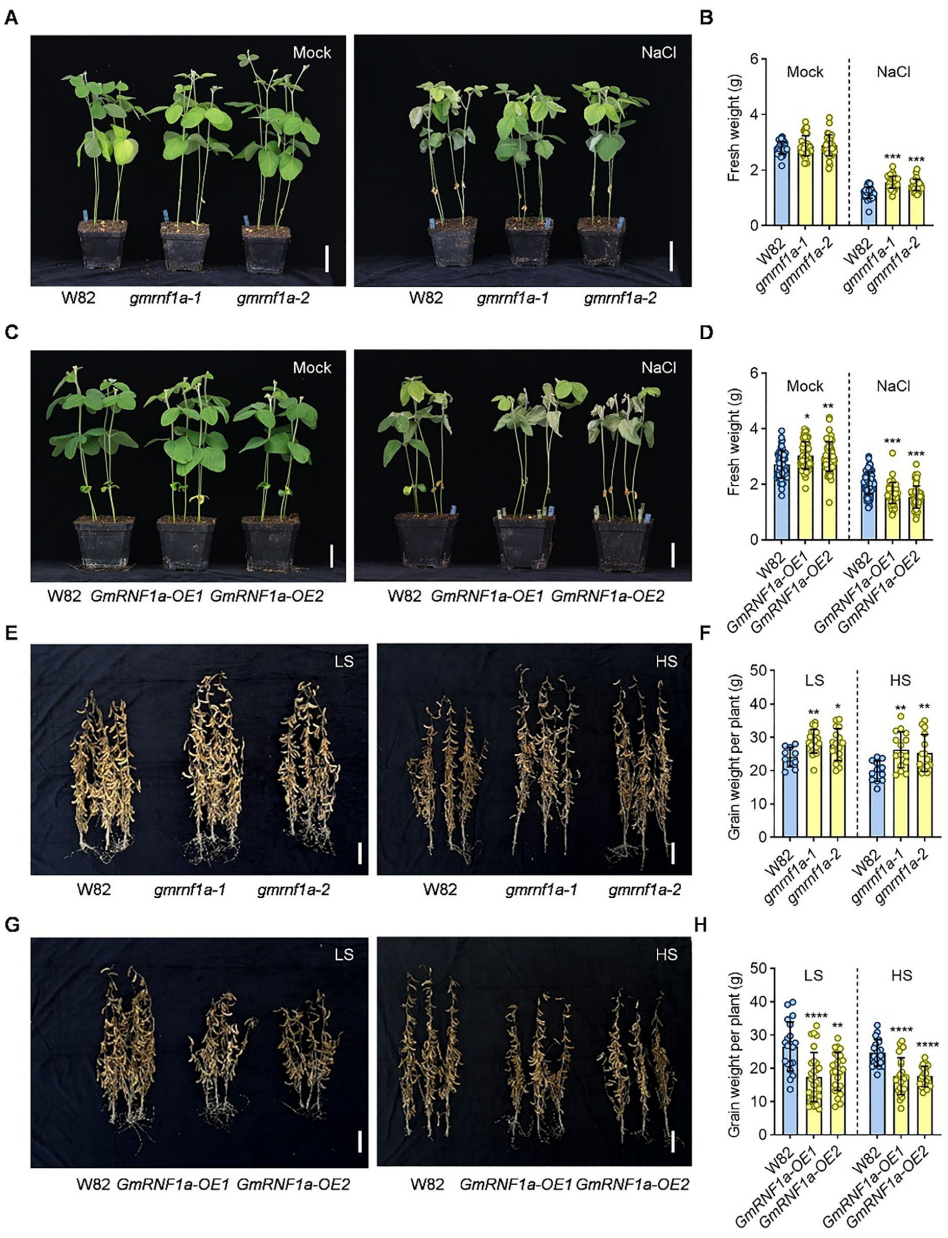
*GmRNF1a* negatively regulates soybean salt tolerance. (A) Salt stress phenotypes of wild‐type (W82) and *gmrnf1a* mutant seedlings after 7 days of 150 mM NaCl treatment. (B) Total fresh weight of seedlings shown in (A). (C) Salt stress phenotypes of W82 and *GmRNF1a‐OE* seedlings after 7 days of 150 mM NaCl treatment. (D) Total fresh weight of seedlings shown in (C). (E) Mature plant phenotypes of W82 and *gmrnf1a* mutants under low‐ and high‐salinity conditions. (F) Grain weight per plant for W82 and *gmrnf1a* mutants under the conditions in (E). (G) Mature plant phenotypes of W82 and *GmRNF1a‐OE* lines under low‐ and high‐salinity conditions. (H) Grain weight per plant for W82 and *GmRNF1a‐OE* lines under the conditions in (G). Scale bar = 4 cm in (A) and (C), 15 cm in (E) and (G). All the data points are shown on the plots. Data in (B) and (D) are presented as mean ± SD (n > 30, 3 independent experiments). All the data points are shown on the plots. Data in (B) and (D) are presented as mean ± SD (n > 30, 3 independent experiments). Data in (F) and (H) are presented as mean ± SD (n > 15, 3 different sites). These data were analyzed by one‐way ANOVA followed by Fisher's LSD test (two‐sided), asterisks indicate significant differences (^*^ *p* < 0.05; ^**^ *p* < 0.01; ^***^ *p* < 0.001; ^****^ *p* < 0.0001).

To explore how GmRNF1a regulates salt tolerance, we generated *GmRNF1a‐OE* transgenic lines in the W82 background by introducing the *35Spro::GmRNF1a* construct. RT‐qPCR revealed a 202‐fold and 152‐fold upregulation of *GmRNF1a* expression in the two independent transgenic lines compared to the wild‐type W82, respectively (Figure S9F). We then examined the phenotype of *GmRNF1a‐OE* transgenic lines under normal conditions. Yield‐related trait analysis showed that, although the plant height of *GmRNF1a‐OE* was slightly higher than that of the wild‐type W82, the number of pods, seeds per plant, and grain weight per plant were significantly reduced (Figure S9A–E). To further investigate the function of *GmRNF1a* in soybean salt tolerance, we characterized the salt tolerance of *gmrnf1a* knockout mutants (*gmrnf1a‐1*, *gmrnf1a‐2*) and *GmRNF1a‐OE* lines (Figure 5A,C). After a 7‐day treatment with 150 mM NaCl under controlled greenhouse conditions, *gmrnf1a‐1* and *gmrnf1a‐2* exhibited less wilting and greater fresh weight than the wild‐type plants (Figure 5A,B), whereas *GmRNF1a‐OE* plants exhibited pronounced wilting and lower fresh weight (Figure 5C,D). To evaluate the salt tolerance of the *gmrnf1a* mutants and *GmRNF1a‐OE* lines under saline soil conditions, we conducted field experiments at two salinity levels. Yield‐related trait analysis showed that, compared to W82, the *gmrnf1a* mutants exhibited significantly increased plant height, pod number, and grain weight per plant in 2025 (Figure 5E,F; Figure S10A–C). In contrast, the *GmRNF1a‐OE* lines displayed a clear salt‐sensitive phenotype, with significant reductions in these traits relative to W82 in both 2024 and 2025 (Figure 5G,H; Figures S9G–L and S10D–F). These contrasting phenotypes confirm that *GmRNF1a* negatively regulates salt tolerance.

To evaluate the performance of the transgenic line across a broader salinity range, we conducted additional salinity gradient experiments (near‐non‐saline (0‐0.1%), moderate‐salinity (0.2%–0.3%), and high‐salinity (0.4%–0.6%)) using soil collected from saline fields in Dongying, China. The results revealed that under near‐non‐saline conditions, there were no significant differences in plant height and pod number per plant among *gmrnf1a*, *GmRNF1a‐OE*, *gmsin1 gmsin1h*, and the wild‐type W82 (Figure S11A,D,E,J,M,N). Under moderate‐salinity conditions, *gmrnf1a* plants exhibited increased plant height and pod number per plant compared to W82 (Figure S11B,F,G), whereas *GmRNF1a‐OE* and *gmsin1 gmsin1h* plants showed significant reduction in both traits (Figure S11B,F,G,K,O,P). Under high‐salinity conditions, *gmrnf1a* plants exhibited significantly increased plant height and pod number per plant compared to W82 (Figure S11C,H,I), whereas *GmRNF1a‐OE* and *gmsin1 gmsin1h* plants showed significant reduction in both traits (Figure S11C,H,I,L,Q,R). The above results further demonstrate the roles of GmRNF1a and GmSIN1 in regulating plant height and pod number per plant under two saline conditions. Notably, the *gmrnf1a* mutant exhibited a salt‐tolerant phenotype across a range of salt concentrations without compromising growth under non‐saline conditions, highlighting its potential as valuable germplasm for breeding salt‐tolerant crops.

### 2.6 *GmSIN1* acts Downstream of *GmRNF1a* to Mediate Salt‐Stress Responses

**FIGURE 6:**
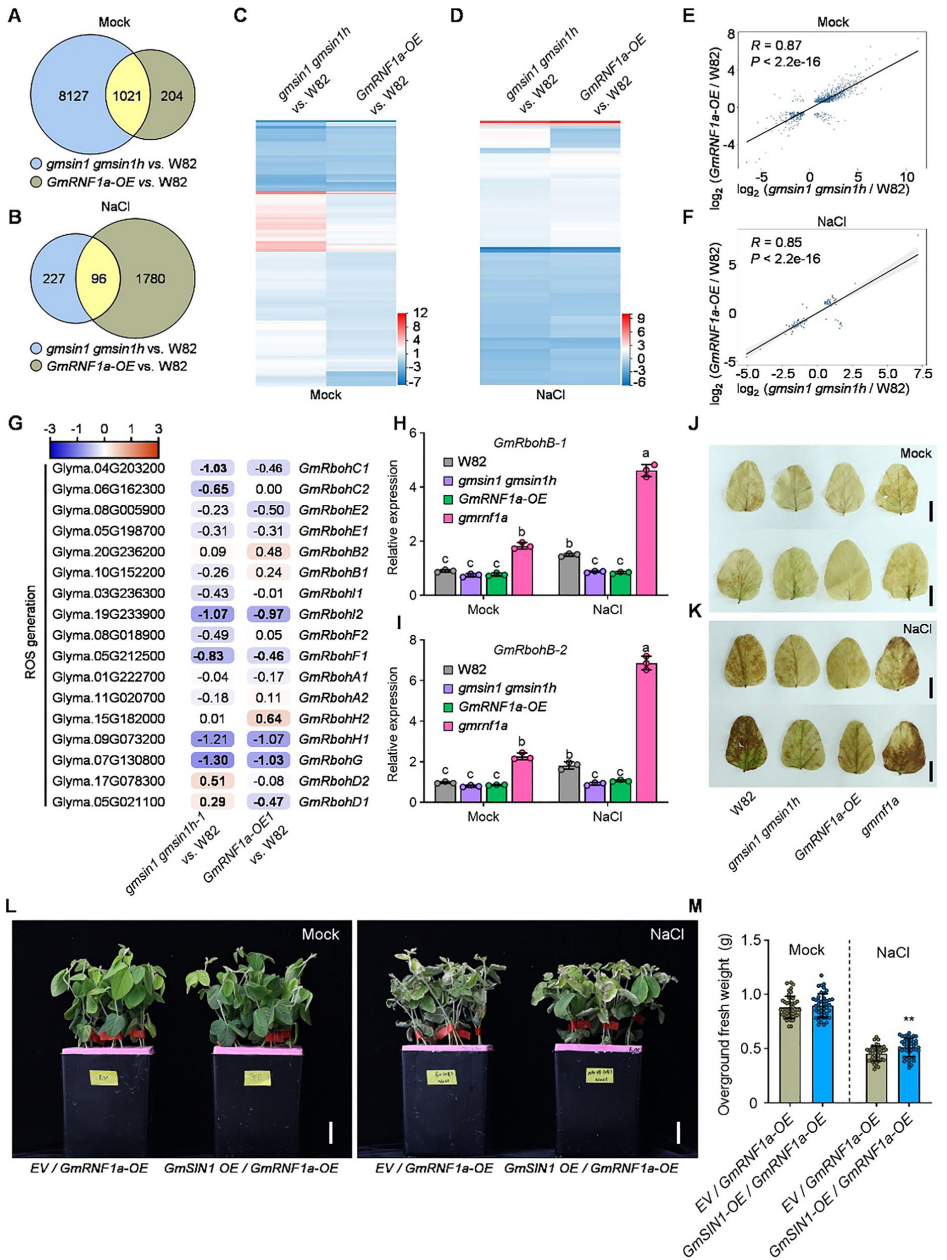
GmSIN1 acts downstream of GmRNF1a in regulating the soybean salt stress response. (A) Venn diagram of differentially expressed genes (DEGs) between *gmsin1 gmsin1h* and *GmRNF1a‐OE* lines compared to W82 under mock conditions. (B) Venn diagram of DEGs between the same genotypes under 150 mM NaCl treatment for 12 h. (C, D) Expression heatmaps of the overlapping DEGs identified in a and b under mock and NaCl conditions, respectively. (E, F) Positive correlation of DEG expression profiles between *gmsin1 gmsin1h* vs. W82 and *GmRNF1a‐OE* vs. W82 under: Normal conditions: R = 0.87 (p < 2.2 × 10−16); Salt stress: R = 0.85 (p < 2.2 × 10−16). (G) Expression heatmaps of DEGs related to ROS generation in *gmsin1 gmsin1h*, and *GmRNF1a‐OE* lines under NaCl stress. (H, I) RT–qPCR validation of *GmRbohB‐1* and *GmRbohB‐2* expression in roots of indicated genotypes under water or NaCl treatment. (J, K) DAB staining for H_2_O_2_ accumulation in seedlings of the indicated genotypes after 12 h NaCl treatment. Scale bar = 2 cm. (L) Phenotypes of composite soybean plants with empty vector (EV) or *GmSIN1‐OE* hairy roots in the *GmRNF1a‐OE1* background after 5 days of NaCl treatment. Scale bar = 3 cm. (M) Overground fresh weight analysis of plants shown in (L). Data in (H)–(I) are presented as mean ± SD (n = 3 biological replicates). These data were analyzed by one‐way ANOVA followed by Tukey's test (two‐sided), different letters indicate significant differences (*p* < 0.05). Data in m are presented as mean ± SD (n > 45, 3 independent experiments). These data were analyzed by *Student t*‐test (two‐side), asterisks indicate significant differences (^*^ *p* < 0.05; ^**^ *p* < 0.01).

To investigate how GmSIN1 and GmRNF1a jointly regulate the salt‐stress response, we performed RNA‐seq on the roots of 14‐day‐old wild‐type W82, *GmRNF1a‐OE1*, and *gmsin1 gmsin1h‐1* plants under control and salt‐stress conditions (150 mM NaCl, 12 h). Under control conditions, compared to the wild type W82, we detected 9,148 differentially expressed genes (DEGs; fold change >2, *p <* 0.05) in *gmsin1 gmsin1h‐1* and 1,225 DEGs in *GmRNF1a‐OE1*, with 1,021 co‐regulated genes overlapping between the genotypes (Figure 6A). Under salt stress, 323 and 1,876 DEGs were identified in *gmsin1 gmsin1h‐1* and *GmRNF1a‐OE1*, respectively, sharing 96 common genes (Figure 6B). To analyze the expression patterns of the DEGs under normal and salt‐stress conditions, we generated a heatmap based on the RNA‐seq data, comparing the transcriptional profiles of DEGs in *gmsin1 gmsin1h‐1* versus W82 and *GmRNF1a‐OE1* versus W82. Notably, DEGs from *gmsin1 gmsin1h‐1* versus W82 and *GmRNF1a‐OE1* versus W82 exhibited consistent up‐ or downregulated expression under both normal and salt‐stress conditions, suggesting that *GmSIN1* and *GmRNF1a* co‐regulate a shared salt tolerance pathway (Figure 6C,D). The expression profiles of all DEGs in *gmsin1 gmsin1h‐1* versus W82 and *GmRNF1a‐OE1* versus W82 were positively correlated under both normal conditions (*R* = 0.87, *p <* 2.2 × 10^−16^) and salt‐stress conditions (*R* = 0.85, *p <* 2.2 × 10^−16^) (Figure 6E,F).

We previously demonstrated that GmSIN1 regulates plant adaptation to salt stress by modulating the expression of genes involved in reactive oxygen species (ROS) generation and abscisic acid (ABA) biosynthesis [27]. To investigate the differential responses of the co‐regulated downstream genes, we analyzed their expression patterns in the RNA‐seq data. Under salt stress (150 mM NaCl, 12 h), both *GmRNF1a‐OE1* and *gmsin1 gmsin1h‐1* exhibited consistent downregulation of ROS‐generation‐related genes (*GmRbohBs*) compared to levels in the wild type W82 (Figure 6G). ABA‐biosynthesis‐related genes (*GmNCED3*) also exhibited lower expression (Figure S12). To validate the transcriptional regulation of the *GmRbohB* homologs (respiratory burst oxidase homologs; *GmRbohB‐1* and *GmRbohB‐2*), we performed RT‐qPCR using RNA from the roots of W82, *GmRNF1a‐OE1*, *gmsin1 gmsin1h‐1*, and *gmrnf1a‐1* plants. *GmRbohB‐1* and *GmRbohB‐2* were slightly downregulated in *GmRNF1a‐OE1* and *gmsin1 gmsin1h‐1* roots compared to the wild‐type W82 under both normal and salt‐stress conditions (Figure 6H,I). Conversely, these genes were markedly upregulated in the *gmrnf1a* mutants (*GmRbohB‐1*: 3.08‐fold, *p <* 0.001; *GmRbohB‐2*: 3.77‐fold, *p <* 0.001) under salt stress (Figure 6H,I). We measured ROS accumulation in these plants using 3,3′‐diaminobenzidine (DAB) staining. Under normal conditions, all genotypes (W82, *GmRNF1a‐OE1*, *gmsin1 gmsin1h‐1*, and *gmrnf1a‐1*) showed comparable basal ROS levels. However, under salt stress (150 mM NaCl, 12 h), *GmRNF1a‐OE1* and *gmsin1 gmsin1h‐1* exhibited lower ROS levels than the wild type W82, whereas *gmrnf1a‐1* had higher ROS levels (Figure 6J,K). These results suggest that GmSIN1 and GmRNF1a jointly regulate the expression of genes involved in ROS generation to help maintain ROS homeostasis.

To explore the genetic relationship between *GmSIN1* and *GmRNF1a* in salt tolerance, we generated transgenic soybean hairy roots in the *GmRNF1a‐OE1* transgenic background harboring either the EV or *UBIpro::GmSIN1‐GFP* construct. RT‐qPCR analysis revealed a 41‐fold upregulation of *GmSIN1* expression in transgenic hairy roots compared to the EV control (Figure S13). After a 5‐day treatment with 150 mM NaCl under controlled greenhouse conditions, transgenic plants overexpressing both *GmSIN1* and *GmRNF1a* displayed less wilting and greater fresh weight than *EV/GmRNF1a‐OE1* plants (Figure 6L,M), demonstrating enhanced salt tolerance. These results indicate that *GmSIN1* is epistatic to *GmRNF1a* in regulating salt tolerance.

### 2.7 *GmCSN5a* Positively Regulates Salt Tolerance in Soybean

**FIGURE 7:**
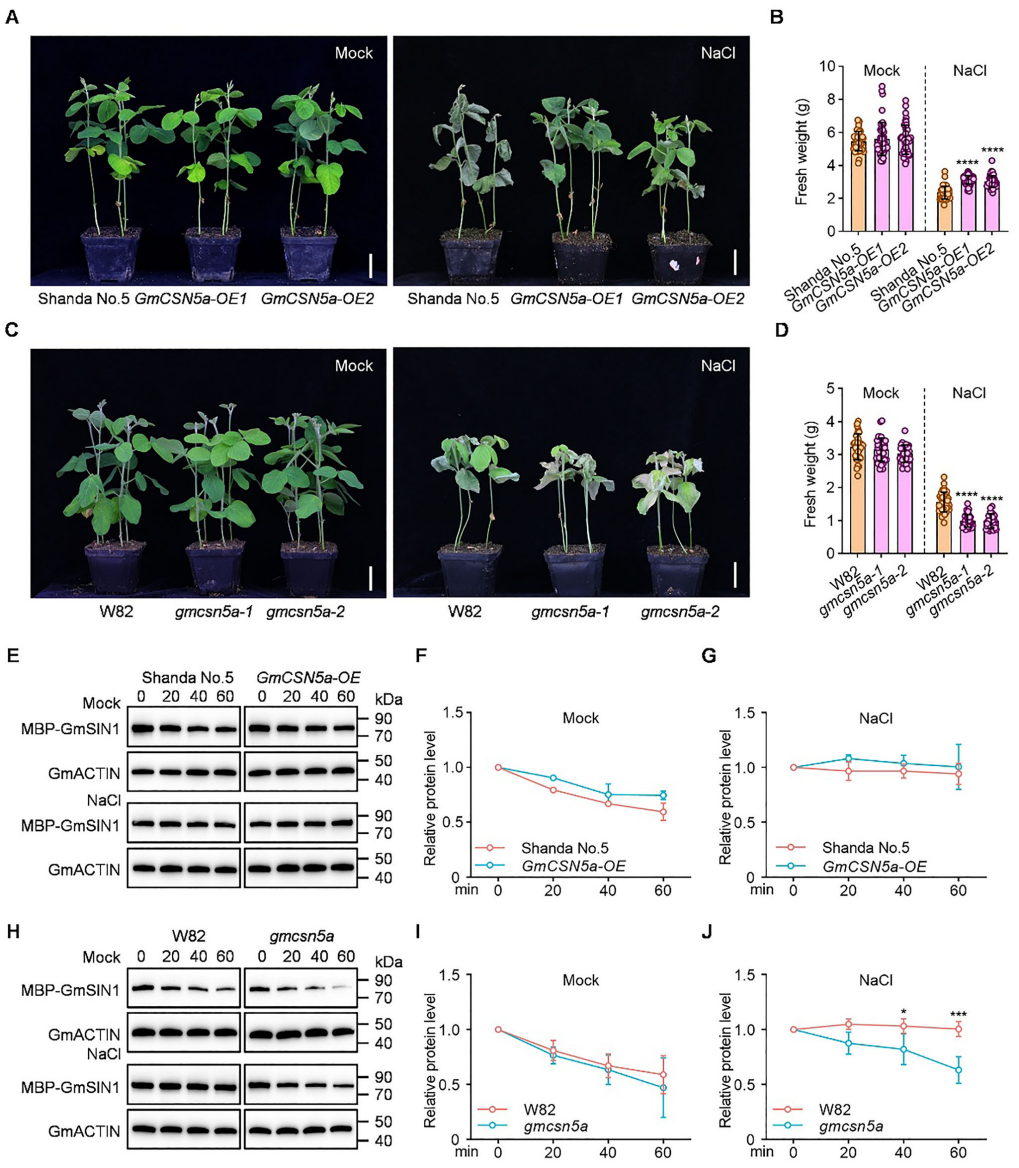
GmCSN5a enhances salt tolerance by stabilizing GmSIN1 protein. (A) Phenotypes of *GmCSN5a‐OE* and wild‐type (Shanda No.5) seedlings under mock or NaCl conditions. Scale bar = 3 cm. (B) Total fresh weight of seedlings shown in (A). (C) Phenotypes of *gmcsn5a* mutants and wild‐type (W82) seedlings under mock or NaCl conditions. Scale bar = 3 cm. (D) Total fresh weight of seedlings shown in (C). (E) In vitro degradation assay of GmSIN1 using root protein extracts from wild‐type and *GmCSN5a‐OE* seedlings with or without prior NaCl treatment. (F, G) Quantification of GmSIN1 band intensity from (E). (H) In vitro degradation assay of GmSIN1 using root protein extracts from wild‐type and *gmcsn5a* seedlings with or without prior NaCl treatment. (I, J) Quantification of GmSIN1 band intensity from (H). Data in (B) and (D) are presented as mean ± SD (n > 36, 3 independent experiments), with all individual data points shown. These data were analyzed by one‐way ANOVA followed by Tukey's test (two‐sided), asterisks indicate significant differences (^****^ *p* < 0.0001). Data in (F), (G), (I), and (J) are presented as mean ± SD (n = 3 biological replicates). Data in (J) were analyzed by *Student t*‐test (two‐side), asterisks indicate significant differences (^*^ *p* < 0.05; ^***^ *p* < 0.001).

To elucidate the role of *GmCSN5a* in salt tolerance, we generated knockout mutants (*gmcsn5a*, T_3_ generation) through CRISPR‐Cas9 genome editing targeting *GmCSN51a* in the W82 background. Two independent lines carried frameshift mutations: an 8‐bp deletion or a 1‐bp insertion in the first exon of GmCSN5a, resulting in premature termination of translation (Figure S14A–C). Meanwhile, we generated *GmCSN5a‐OE* transgenic lines in the Shanda No.5 background. RT‐qPCR revealed a 350‐fold and 262‐fold upregulation of *GmCSN5a* expression in the two independent transgenic lines compared to the wild‐type Shanda No.5, respectively (Figure S14D). We then characterized the salt tolerance of *gmcsn5a* knockout mutants (*gmcsn5a‐1*, *gmcsn5a‐2*) and *35Spro::GmCSN5a‐OE* transgenic soybean lines (Figure 7A,C). After treated with 150 mM NaCl, *GmCSN5a‐OE* plants exhibited less wilting and higher fresh weight than the wild‐type plants (Figure 7A,B), whereas *gmcsn5a* plants exhibited pronounced wilting and lower fresh weight (Figure 7C,D). These results establish that *GmCSN5a* is a positive regulator of salt tolerance in soybean.

To further investigate the role of GmCSN5a in the degradation of GmSIN1, we performed cell‐free degradation assays using total protein extracts from wild‐type Shanda No. 5 and *GmCSN5a‐OE* roots. MBP‐GmSIN1 exhibited slightly slower time‐dependent degradation when incubated with *GmCSN5a‐OE* roots extract compared to wild‐type Shanda No. 5; however, this difference in degradation was not observed in reactions containing salt‐treated roots (Figure 7E–G). Furthermore, we found that the time‐dependent degradation of GmSIN1 was similar when incubated with W82 and *gmcsn5a*. Notably, MBP‐GmSIN1 exhibited accelerated time‐dependent degradation when incubated with NaCl‐treated *gmcsn5a* roots (Figure 7H–J), indicating that GmCSN5a is a critical regulator for inhibiting GmSIN1 degradation during salt stress.

### 2.8 Haplotypes Analysis of *GmSIN1*, *GmRNF1a*, and *GmCSN5a* in Soybean Accessions

To investigate natural variation in *GmSIN1*, *GmRNF1a*, and *GmCSN5a* across natural soybean populations, we performed haplotype‐based analyses of SNPs and InDels within their coding regions and promoters. Analyzing resequencing data from 592 soybean accessions, distinct major haplotypes were identified for each gene: *GmSIN1* exhibited two haplotypes (n = 523; Hap1 = 482, Hap2 = 41), *GmRNF1a* displayed three (n = 508; Hap1 = 324, Hap2 = 109, Hap3 = 75), and *GmCSN5a* showed two (n = 481; Hap1 = 268, Hap2 = 213) (Figure S15A–C). Haplotype network analysis revealed that *GmSIN1^Hap1^* and *GmSIN1^Hap2^* are closely related, differing by only one SNP, indicating *GmSIN1^Hap2^* likely originated from *GmSIN1^Hap1^*. In contrast, the three *GmRNF1a* haplotypes showed relatively distant genetic relationships. For *GmCSN5a*, Hap2 and Hap3 formed a close cluster, while Hap1 was more distantly related (Figure S16A–C).

Furthermore, we investigated the association between dominant haplotypes in cultivated soybean varieties and artificial selection during domestication. This analysis revealed that the dominant haplotypes of *GmSIN1*, *GmRNF1a*, and *GmCSN5a* were predominant in cultivated soybeans. Notably, the frequencies of *GmSIN1^Hap2^*, *GmRNF1a^Hap3^*, *GmCSN5a^Hap1,^* and *GmCSN5a^Hap2^* consistently increased from wild soybeans (*G. soja*) to landraces to elite cultivars (Figure S15D–F), suggesting positive selection of these haplotypes during soybean domestication and modern breeding.

### 2.9 Identification of Elite Haplotypes in *GmSIN1*, *GmRNF1a*, and *GmCSN5a*

**FIGURE 8:**
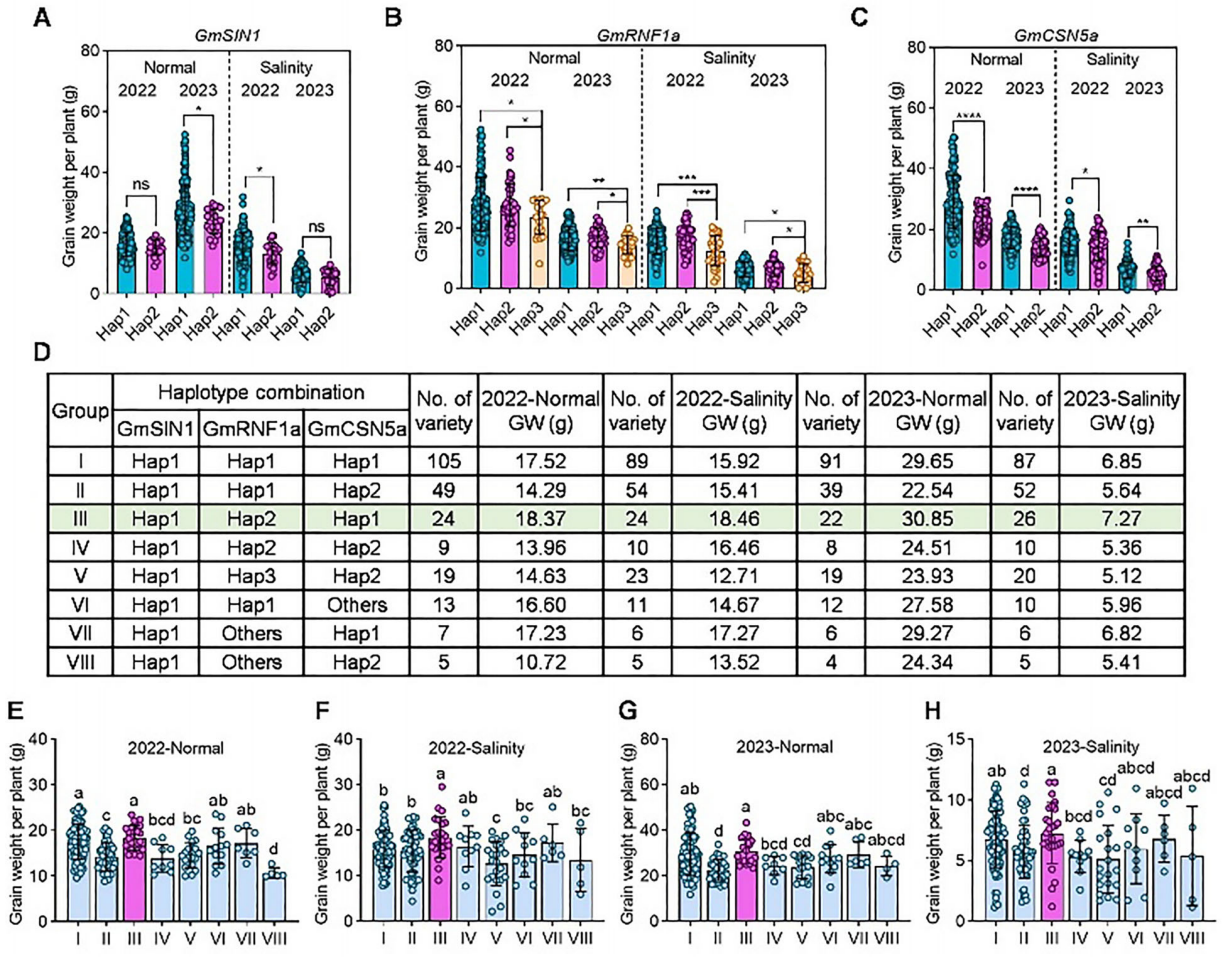
Combined haplotype analysis of *GmSIN1*, *GmRNF1a*, and *GmCSN5a* reveals breeding potential for grain yield and salt tolerance. (A–C) Grain weight per plant for the main haplotypes of *GmSIN1*, *GmRNF1a*, and *GmCSN5a* under normal and saline conditions across two growing seasons (2022 and 2023). (D) Performance of combined haplotypes of *GmSIN1*, *GmRNF1a*, and *GmCSN5a* under normal and saline conditions.GW, Grain weight per plant. The green box highlights the haplotype combination with the highest grain yield among the eight tested combinations. (E–H) Grain weight per plant for different haplotype combinations under normal and saline conditions across the 2022 and 2023 growing seasons. All the data points are shown on the plots. Data in (A)–(C) are presented as mean ± SD (n ≥ 27). Data in (A) and (C) were analyzed by *Student t*‐test (two‐side), asterisks indicate significant differences (^*^ *p* < 0.05; ^**^ *p* < 0.01; ^***^ *p* < 0.001; ^****^ *p* < 0.0001). Data in (B) were analyzed by one‐way ANOVA followed by Fisher's LSD test (two‐sided), asterisks indicate significant differences (^*^ *p* < 0.05; ^**^ *p* < 0.01; ^***^ *p* < 0.001). Data in (E)–(H) are presented as mean ± SD (n ≥ 4). These data were analyzed by one‐way ANOVA followed by Fisher's LSD test (two‐sided), different letters indicate significant differences (*p* < 0.05).

To investigate the impact of haplotypes on soybean yield, we performed association analyses between major haplotypes of *GmSIN1*, *GmRNF1a*, and *GmCSN5a* and yield‐related traits. These analyses were conducted under both normal and saline conditions with a population of 440 accessions and a population of 250 accessions, respectively. Significant differences in grain weight per plant were observed among the haplotypes for all three genes. Specifically, *GmSIN1^Hap1^* showed significantly higher grain weight than *GmSIN1^Hap2^* under normal conditions in 2023 and under saline conditions in 2022, with a similar trend under normal conditions in 2022 and saline conditions in 2023 (Figure 8A). For *GmRNF1a*, while no significant difference in grain weight was found between Hap1 and Hap2, both haplotypes significantly outperformed Hap3 under normal conditions and under saline conditions in 2022 and 2023 (Figure 8B). Similarly, *GmCSN5a^Hap1^* demonstrated significantly greater grain weight than *GmCSN5a^Hap2^* under normal conditions and under saline conditions in 2022 and 2023 (Figure 8C). Collectively, these results indicate that elite haplotypes that enhance soybean yield under both normal and saline conditions exist within the salt‐tolerance‐associated genes *GmSIN1*, *GmRNF1a*, and *GmCSN5a*.

To verify the consistency of the elite haplotype's effects across different genetic backgrounds, we classified the 592 germplasms into three main ecotypes based on geographic origin: Northern Spring Soybean, Huang‐Huai‐Hai Summer Soybean, and Southern Double‐Cropped Soybean. We conducted haplotype association analyses within each ecotype (except Southern Double‐Cropped Soybean, due to its limited number of accessions) using saline field data of two years. In the Northern Spring Soybean populations, plants harboring the elite haplotypes of *GmSIN1*, *GmCSN5a*, or *GmRNF1a* had significantly higher grain weight per plant compared to plants with other haplotypes in 2022, and also showed an increasing trend in 2023 (Figure S17A–C). In the Huang‐Huai‐Hai Summer Soybean population, plants harboring the elite haplotype of *GmSIN1* consistently showed significantly higher grain weight per plant in both years, while plants harboring the elite haplotypes of *GmCSN5a* and *GmRNF1a* exhibited similar increasing trends (Figure S17D–F). This result suggests that the elite haplotypes of these genes are promising for breeding applications in both the Northern Spring and Huang‐Huai‐Hai Summer soybean varieties.

### 2.10 Pyramiding Elite Haplotypes of *GmSIN1*, *GmRNF1a*, and *GmCSN5a* Improves Soybean Yield

We further investigated the pyramiding effects of the elite haplotypes on soybean yield under normal and saline conditions. The result showed that pyramiding the three elite haplotypes in Combination III (*GmSIN1^Hap1^*‐*GmRNF1a^Hap2^*‐*GmCSN5a^Hap1^*) resulted in the highest grain weight per plant among all combinations under normal and saline conditions in 2022 and 2023 (Figure 8D). In 2022, Combination III exhibited significantly higher grain weight per plant than Combinations (II, IV, V, and VIII) in the normal field and Combinations (I, II, V, VI, and VIII) in the saline field, respectively (Figure 8E,F). In 2023, Combination III exhibited significantly higher grain weight per plant than Combinations (II, IV, and V) in the normal field and Combinations (II, IV, and V) in the saline field, respectively (Figure 8G,H). Notably, cultivars harboring Combination III comprised only one‐fifth of the top 50 accessions ranked by grain weight under saline conditions (Figure S18A,B), indicating substantial potential for pyramiding these elite haplotypes to enhance soybean yield in saline soil. Collectively, pyramiding elite haplotypes of *GmSIN1*, *GmRNF1a*, and *GmCSN5a* provides a novel strategy for improving soybean yield under both normal and saline fields.

To further investigate the underlying mechanisms of how different haplotype combinations affect soybean yield under saline soil conditions, we first analyzed the impact of these haplotypes on protein structures. The *GmSIN1* and *GmCSN5a* haplotypes are primarily located in the promoter region, which is hypothesized to affect their transcription (Figure S15A,C). A 6‐bp indel exists between GmRNF1a haplotypes 1/3 and haplotype 2, resulting in the insertion of two amino acids into the GmRNF1a protein (Figure S19A). Using AlphaFold3 for structural prediction and PyMOL for visualization, we found that this insertion alters the overall conformation of the GmRNF1a protein without affecting its core C2H2 domain structure (Figure S19B). We identified three potential ubiquitination sites on GmSIN1 (K62, K96, K102) using the GPS‐Uber Web Server. PyMOL analysis revealed that the conformational change in GmRNF1a increases the distance between its catalytic core and GmSIN1 ubiquitination sites, resulting in weaker ubiquitination of GmSIN1 by GmRNF1a^Hap2^ compared to GmRNF1a^Hap1^ (Figure S19C,D). Furthermore, we analyzed the protein conformations of GmCSN5a in complex with different GmRNF1a haplotypes. The distance between the key domain (MPN) of GmCSN5a and the active center of GmRNF1a was shorter in the GmRNF1a^Hap2^‐GmCSN5a complex than in the GmRNF1a^Hap1^‐GmCSN5a complex (Figure S19E,F), suggesting that GmCSN5a may most strongly inhibit the E3 ligase activity of GmRNF1a^Hap2^. Additionally, when all three proteins were present, the distance between the active center of GmRNF1a^Hap2^ and GmSIN1 ubiquitination sites was greater in the GmSIN1‐GmRNF1a^Hap2^‐GmCSN5a complex compared to the GmSIN1‐GmRNF1a^Hap1^‐GmCSN5a complex (Figure S19G,H).

## 3 Discussion

The response of soybean to salt stress entails complex molecular networks and multiple physiological and biochemical coping mechanisms [1, 3]. Although more than 100 salt tolerance genes in soybean have been functionally characterized [10, 42, 43], the breeding of salt‐tolerant cultivars remains constrained by limited genetic resources, and their molecular mechanisms at the protein level remain poorly understood [9]. In this study, we demonstrated that GmRNF1a and GmCSN5a jointly regulate the stability of GmSIN1 through ubiquitination under salt stress. Importantly, the haplotypes of *GmRNF1a*, *GmSIN1*, and *GmCSN5a* provide a natural variation source for salt‐tolerant soybean breeding. Pyramiding these elite haplotypes can elevate grain weight per plant in soybean accessions, accelerating the pace of cultivar development.

### 3.1 Two Novel Genes (GmRNF1a and GmCSN5a) Regulate Salt Tolerance and Yield in Soybean

Soybean (Glycine max), a crucial food and oil crop, faces severe production constraints due to salt stress. Identifying salt‐tolerance genes and elucidating their mechanisms are essential for improving soybean resilience and yield. While GmRNF1a was previously implicated in seed oil content and pod dehiscence in Arabidopsis [44, 45], its role in soybean salt tolerance remained unknown. Here, we found that the *gmrnf1a* mutants were salt‐tolerant, whereas the *GmRNF1a‐OE* lines exhibited decreased salt tolerance (Figure 5). This indicates that GmRNF1a acts as a negative regulator of salt tolerance and yield. Additionally, GmCSN5a, a core subunit of the COP9 signalosome, was previously linked to salt stress in Arabidopsis [46], but its function and molecular mechanism in soybean salt tolerance remained unclear. Here, we found that the *GmCSN5a‐OE* lines were salt‐tolerant, whereas the *gmcsn5a* mutants exhibited decreased salt tolerance (Figure 7A–D). This indicates that *GmCSN5a* acts as a positive regulator of salt tolerance in soybean. Collectively, our study identifies GmRNF1a and GmCSN5a as novel regulators of soybean salt tolerance and yield, providing mechanistic insights that expand the salt‐stress regulatory network and offer a theoretical foundation for breeding salt‐tolerant soybean varieties.

### 3.2 GmCSN5a Directly Inhibits E3 Ligase Activity of GmRNF1a Independently of CSN‐CRL Complexes

Protein homeostasis (proteostasis) maintains cellular function and survival through the precise regulation of protein biosynthesis, post‐translational modifications, and degradation [47]. Ubiquitination, a crucial post‐translational modification, dynamically regulates protein turnover and activity to participate in various physiological processes [28]. The ubiquitination process is collaboratively mediated by the E1‐E2‐E3 enzyme complex, with substrate specificity determined by either E3 ubiquitin ligase monomers or the F‐box subunits within E3 ubiquitin ligase complexes [29]. Plant genomes encode a limited number of E1 and E2 enzymes but a diverse array of E3 ligases [30]. Here, we found that GmRNF1a is a RING‐type E3 ubiquitin ligase; its intrinsic E3 ligase activity was depended on four amino acids (C2H2) in the catalytic core (Figure S4A,B).

The COP9 signalosome (CSN) is an evolutionarily‐conserved protein complex in plants, animals, and yeast, comprising eight subunits (CSN1‐CSN8) [37, 38]. Among these components, CSN5 is a JAMM‐domain metalloprotease (JAB1/MPN/MOV34 family) [37, 39]. CSN5a inhibits E3 ubiquitin ligase activity by removing the NEDD8 modification from the Cullin1 subunit of CRLs (Cullin‐RING E3 ligase complex) [40, 41]. These mechanisms by which CSN‐CRL complexes regulate the activity of E3 ubiquitin ligases through deneddylation have been extensively studied [37]. However, no studies have found that GmCSN5a modulates E3 ubiquitin ligase activity through direct interaction in a CSN‐CRL complexes‐independent manner. Here, we found that GmCSN5a directly interacts with GmRNF1a through its zinc ribbon domain (Figure 4A,B). Furthermore, GmCSN5a itself directly inhibits the E3 ligase activity of GmRNF1a through its MPN domain (Figure S7C). In addition, GmRNF1a is not a member of the CRL family and possesses intrinsic E3 ubiquitin ligase activity. Therefore, our findings reveal a new mechanism in which CSN5 directly inhibits the E3 ligase activity of GmRNF1a, independent of the conventional CSN‐CRL complex‐dependent pathway [48]. Future research should explore the molecular basis of GmCSN5a‐mediated inhibition of the E3 ligase activity of GmRNF1a.

### 3.3 The GmSIN1‐GmRNF1a‐GmCSN5a Module Regulates Salt Tolerance by Maintaining Protein Homeostasis

**FIGURE 9:**
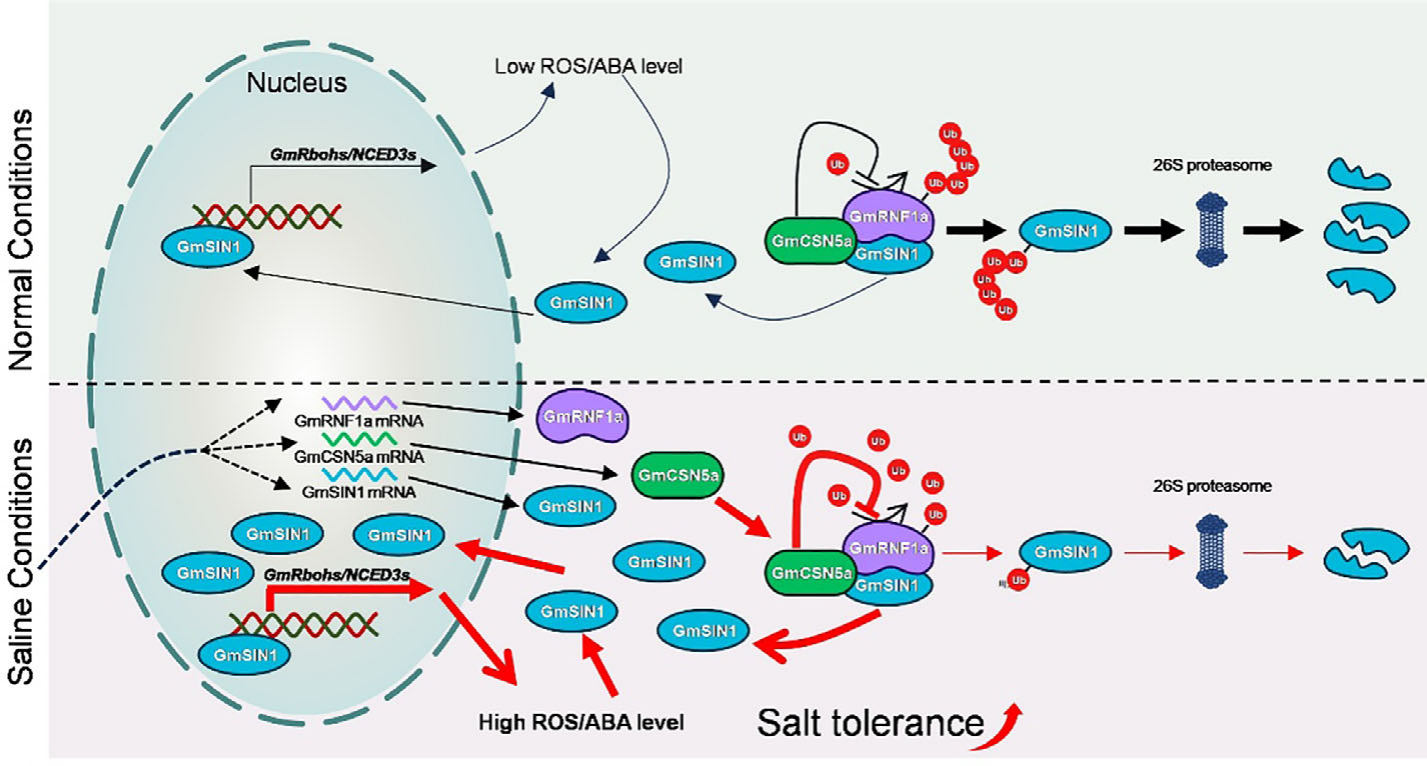
A working model of GmSIN1‐GmRNF1a‐GmCSN5a in regulating salt tolerance in soybean. Under normal conditions, GmRNF1a (purple irregular shape) ubiquitinates GmSIN1 (cyan ellipse), targeting it for degradation via the 26S proteasome to maintain low cellular levels. GmCSN5a (green rounded rectangle) constitutively inhibits GmRNF1a's E3 ligase activity, and this antagonism, together with low GmSIN1 transcription, ensures its minimal expression. Under salt stress, the model is enhanced through two coordinated mechanisms: (i) transcriptional induction of *GmSIN1*, *GmRNF1a*, and *GmCSN5a*; (ii) salt‐induced accumulation of GmCSN5a more potently suppresses GmRNF1a activity, leading to reduced GmSIN1 ubiquitination and degradation. The combined transcriptional and post‐translational regulation enables rapid GmSIN1 protein accumulation. Subsequently, GmSIN1 activates downstream targets (e.g., *GmRbohs* and *GmNCED3s*) to drive the ROS/ABA pathway and improve salt tolerance. Graphical notation: red solid arrows indicate strong activation; black arrows indicate weak promotion; red T‐bar arrowheads indicate strong inhibition; black T‐bar arrowheads indicate weak inhibition.

Plants respond to abiotic stress through intricate gene regulatory networks [2]. Salt stress triggers multi‐layered adaptive mechanisms by integrating osmotic, ionic, ROS, and phytohormone (e.g., ABA, ethylene, jasmonic acid) signaling pathways [6, 25]. TFs act as central hubs, coordinating these signals to precisely regulate downstream gene expression and mediate transitions between normal growth and stress adaptation [14, 15]. Consequently, TF expression must be tightly controlled. While TF regulation at the RNA level has been extensively studied, for example, most GmNTL family members transcriptionally respond to salt and other abiotic stresses [27], protein‐level regulation, particularly ubiquitination‐mediated proteostasis, remains underexplored in soybean. A notable exception is GmCHYR16, an E3 ubiquitin ligase that regulates bicarbonate stress responses by ubiquitinating and degrading the TF GmERF71 [34]. Here, we found that GmRNF1a is a negative regulator of soybean salt tolerance, which ubiquitinates GmSIN1 and promotes its degradation (Figures 3 and 5). This further highlights the crucial role of 26S proteasome‐mediated degradation in the precise regulation of TF homeostasis for environmental adaptation. Besides, GmRNF1a interacts with both the MPN and ICA domains of GmCSN5a via its N‐terminal zinc ribbon domain (Figure 4A,B). GmCSN5a directly inhibits the E3 ligase activity of GmRNF1a, thereby attenuating the ubiquitination of GmSIN1 (Figure 4E–G). Furthermore, GmCSN5a is required for stabilization of GmSIN1 by inhibiting its degradation under salt stress (Figure 7E–J). Based on these findings, we propose a working model illustrating how the ubiquitin‐proteasome pathway regulates GmSIN1 homeostasis. Under normal conditions, GmSIN1 is ubiquitinated by GmRNF1a and degraded via the 26S proteasome pathway, and GmCSN5a inhibits this process. The antagonistic interaction between GmRNF1a and GmCSN5a in maintaining GmSIN1 proteostasis, together with the low transcriptional level of *GmSIN1*, collectively maintains the lower accumulation of GmSIN1 under normal conditions. Under salt stress, *GmSIN1*, *GmRNF1a*, and *GmCSN5a* were transcriptionally induced. GmCSN5a increased more strongly inhibits the E3 ligase activity of GmRNF1a, consequently, the ubiquitination of GmSIN1 by GmRNF1a is reduced, which in turn promotes the accumulation of GmSIN1. The combined effect of transcriptional and post‐translational regulation enables the rapid accumulation and maintenance of the GmSIN1, which subsequently activates the downstream ROS/ABA pathway to improve salt tolerance (Figure 9). Collectively, the GmSIN1‐GmRNF1a‐GmCSN5a module refines the pathway of GmSIN1‐mediated salt tolerance by precisely regulating proteostasis, highlighting the essential role of ubiquitination in modulating TF activity.

### 3.4 Pyramiding Elite Haplotypes of *GmSIN1*, *GmRNF1a*, and *GmCSN5a* Confers High Yield in Soybean

Soybean germplasm resources exhibit rich genetic diversity, suggesting that natural genetic variations (SNPs, Indels, and structural variations) play important roles in trait formation in this crop. Identifying and characterizing natural genetic variants associated with salt tolerance in soybean could facilitate the development of improved varieties for saline soil [9]. To date, the elite haplotypes of five soybean genes associated with improved salt tolerance under controlled greenhouse conditions have been reported [7, 9, 10, 11, 12]. However, the natural haplotypes associated with higher salinity tolerance in field‐grown soybeans under natural conditions remain poorly characterized. In this study, we identified elite haplotypes from *GmSIN1*, *GmRNF1a*, and *GmCSN5a*, respectively (Figure S15). These elite haplotypes were associated with grain weight per plant under both normal and saline soil conditions, demonstrating that pyramiding the *GmSIN1*‐*GmRNF1a*‐*GmCSN5a* elite haplotypes holds great potential for promoting yield in soybean breeding (Figure 8; Figure S17).

Advances in sequencing technologies have enabled high‐quality reference genomes for numerous crops, including soybean (*Glycine max*), facilitating the identification of salt‐tolerance regulatory genes. Multiple salt‐tolerance‐associated quantitative trait loci (QTLs) such as *GmSAL3*, *GmCDF1*, and *GmGI* have been identified and functionally validated in soybean. Nevertheless, research on improving soybean salt tolerance through haplotype pyramiding remains unreported. While elite haplotypes of *GmSIN1* and *GmCSN5a* occur at high frequencies in cultivated soybeans, the *GmRNF1a* elite haplotype (Hap2) shows no evidence of domestication‐driven selection (Figure S15D–F). Furthermore, cultivars pyramiding all three elite haplotypes constitute only a minor fraction of salt‐tolerant accessions, indicating substantial potential for genetic improvement through targeted pyramiding (Figure S19A,B). Critically, pyramiding elite haplotypes of *GmSIN1^Hap1^*, *GmRNF1a^Hap2^*, and *GmCSN5a^Hap1^* confers significant yield advantages under normal and saline field conditions (Figure 8; Figure S17). This may result from the elite haplotypes of *GmRNF1a* and *GmCSN5a* introducing coding‐region variations that increase the spatial distance between *GmSIN1* ubiquitination sites and the *GmRNF1a* active center within the *GmSIN1^Hap1^*‐*GmRNF1a^Hap2^*‐*GmCSN5^Hap1^* module (Figure S19). The increased distance disrupts GmSIN1 ubiquitination by GmRNF1a, thereby stabilizing the protein. This synergistic effect validates haplotype pyramiding as an effective strategy for enhancing soybean yield under salt stress, providing both natural targets and a theoretical foundation for multi‐gene breeding. Collectively, these findings establish a novel breeding paradigm: leveraging pyramided natural variation across multiple genes to simultaneously improve soybean salt tolerance and yield.

In summary, we discovered that the GmSIN1‐GmRNF1a‐GmCSN5a module regulates salt tolerance in soybean by modulating TF ubiquitination, uncovering a previously uncharacterized mechanism by which GmCSN5a suppresses monomeric E3 ligase activity and identifying superior haplotypes of *GmSIN1* and *GmRNF1a*. These findings expand the regulatory hierarchy of salt‐stress signaling, enhance our understanding of eukaryotic ubiquitination mechanisms. Furthermore, the elite haplotypes of *GmSIN1*, *GmRNF1a*, and *GmCSN5a* enrich the soybean genetic resources of and provide both potential targets for genetic improvement and novel strategies for breeding elite salt‐tolerant cultivars.

## 4 Experimental Section

### 4.1 Plant Materials and Growth Conditions

Transgenic Soybean (Glycine max) materials included transgenic and mutant lines in the genetic background of cultivar Williams 82 (W82) or Shanda No.5. The *GmRNF1a‐OE* and *GmCSN5a‐OE* lines were generated via *Agrobacterium tumefaciens*‐mediated cotyledonary‐node transformation in our laboratory. The *gmsin1 gmsin1h*, *gmrnf1a*, and *gmcsn5a* knockout mutants were developed using CRISPR–Cas9 technology (Wuhan Boyuan Biotechnology Co., Ltd.).

For greenhouse cultivation, plants were grown in square pots containing a 1:1 (v/v) mixture of Pindstrup substrate and vermiculite, under controlled conditions of 25°C with a 16‐h light/8‐h dark photoperiod. For salt‐stress phenotyping, plants were cultivated until the V1 stage and then treated with 150 mM NaCl solution for 7 or 14 days. Field trials were conducted in saline soil with two salinity gradients: low salinity (LS, 0.2–0.3 g total soluble salts per 100 g dry soil) and high salinity (HS, 0.3–0.4 g total soluble salts per 100 g dry soil). Salinity gradient treatments were established at three levels: 0‐0.1% (non‑saline control), 0.2‐0.3% (moderate salinity), and 0.4‐0.6% (high salinity). Soil was collected from saline fields in Dongying, China. Plants were grown under these salinity regimes in a controlled‑climate chamber with conditions otherwise maintained uniformly across treatments.

Transgenic soybean hairy roots (genetic backgrounds: W82 or *GmRNF1a‐OE1*) were generated via *Agrobacterium rhizogenes* K599‐mediated transformation and cultivated hydroponically in the greenhouse. Roots were preliminarily screened using a LUYOR‐3415RG fluorescence imaging system (365/470 nm excitation) to select DsRED2‐positive transformants prior to phenotypic analysis. Phenotypes and fresh weights were recorded 4 days post‐treatment.

Tobacco (*Nicotiana benthamiana*) plants were cultivated under greenhouse conditions at 25°C with a 12‐h light/12‐h dark photoperiod.

### 4.2 Vector Construction and Transformation

Fusion constructs (MBP‐GmSIN1, BD‐GmSIN1^1–221^, GmSIN1‐cYFP, GmSIN1‐MYC, GmRNF1a‐nYFP, GST‐GmRNF1a, GST‐GmRNF1a^4Y^, BD‐GmRNF1a, BD‐GmRNF1a^1–76^, BD‐GmRNF1a^77–233^, AD‐GmCSN5a^1–230^, AD‐GmCSN5a^231–375^, GmCSN5a‐nYFP, GST‐GmCSN5a, GmCSN5a‐cYFP, GmCSN5a‐MYC, MBP‐GmCSN5a, MBP‐GmCSN5a^1–230^, MBP‐GmCSN5a^231–375^, and MBP‐GmCSN5a^Δ78–215^) were constructed by directional cloning using the ClonExpress II One‐Step Cloning Kit (Vazyme, C112).

Gateway‐based vectors (GmSIN1‐GFP, GmRNF1a‐GFP, GmCSN5a‐GFP) were generated via BP/LR recombination. Target sequences were first subcloned into the *pDONR22*1 entry vector and subsequently transferred into destination vectors. All primers used for vector construction are listed in Table S1.

### 4.3 RNA Extraction and RT–qPCR

Total RNA was extracted from plant tissues using TRIzol reagent (Mei5bio, MF034). First‐strand cDNA was synthesized from 1 µg of total RNA using HiScript III RT SuperMix (Vazyme, R433‐01), following the manufacturer's instructions. Quantitative PCR was performed on an Applied Biosystems 7500 Real‐Time PCR System using 2× M5 Hipper plus Taq HiFi PCR mix (Mei5bio, MF797‐01). Relative gene expression was calculated via the 2^ΔΔCt^ method, with *GmACTIN* (*GmACT11*, *Glyma.02G091900*) serving as the internal control. All primer sequences are listed in Table S1.

### 4.4 Yeast Two‐Hybrid Assay

Yeast strain Y2HGold was co‐transformed with pairwise combinations of the following constructs: BD‐SIN1^1–221^, BD‐RNF1a, BD‐GmRNF1a^1–76^, BD‐GmRNF1a^77–233^, AD‐CSN5a^1–230^, and AD‐CSN5a^231–375^. Transformants were selected on SD/–Trp/–Leu agar plates and incubated at 28 °C for 96 h. Positive colonies were serially diluted (tenfold steps) in sterile PBS and spotted onto quadruple‐dropout medium (SD/–Ade/–His/–Trp/–Leu) for interaction screening (48 h, 28 °C). Protein–protein interactions were visualized and documented using a GelDoc XR+ imaging system.

### 4.5 Bimolecular Fluorescence Complementation

BiFC vectors (SIN1‐cYFP, RNF1a‐nYFP, CSN5a‐nYFP, CSN5a‐cYFP) were introduced into *Agrobacterium rhizogenes* K599 via the freeze‑thaw transformation method. Transgenic soybean hairy roots were generated as described above and imaged using a Zeiss LSM 880 confocal microscope. YFP fluorescence was detected with excitation at 514 nm and emission collected between 527–553 nm.

### 4.6 GST Pull‐Down Assay

GST pull‐down assays were performed with modifications to established protocols. Recombinant GST‐GmRNF1a and GST‐GmCSN5a proteins were affinity‐purified using GST‐tag Purification Resin (BeyoGold P2251). MBP‐GmSIN1 was isolated with Anti‐MBP Magnetic Beads (BeyoGold P212333). Glutathione agarose‐immobilized bait proteins (GST‐GmRNF1a or GST‐GmCSN5a) were incubated with prey protein (MBP‐GmSIN1 or MBP‐CSN5a) in binding buffer at 4 °C for 2 h. Beads were washed three times with PBS‐T (0.2% Tween‐20) and centrifuged at 15,000 × g for 5 min per wash. Protein complexes were resolved by SDS–PAGE (Epizyme PG212) and immunoblotted with monoclonal anti‐GST (Proteintech 66001‐2‐Ig, 1:5,000) and polyclonal anti‐MBP (Proteintech 15089‐1‐AP, 1:5,000) antibodies.

### 4.7 In Vitro Ubiquitination Assay

Ubiquitination assays were conducted as described [45] with modifications. Standard reactions contained 50 ng recombinant human E1 (rhUBE1; R&D Systems E‐305‐025), 100 ng E2 (rhUbcH5b/UBE2D2; R&D Systems E2‐622‐100), 1 µg Flag‐ubiquitin (R&D Systems U123330), 2 µg GST‐RNF1a (or GFP bead‐enriched GmRNF1a‐GFP from tobacco), and 2 µg MBP‐GmSIN1 in ubiquitination buffer (50 mM Tris‐HCl pH 7.5, 10 mM MgCl_2_, 5 mM ATP, 2 mM DTT). Reactions proceeded at 28 °C for 2 h with shaking, terminated with 5× loading buffer, denatured at 95 °C for 10 min, and analyzed by immunoblotting using anti‐GST (1:5,000), anti‐MBP (1:5,000), and anti‐ubiquitin (Proteintech 10201‐2‐AP, 1:5,000) antibodies. For assessing the effect of GmCSN5a, GmRNF1a‐GFP was immunoprecipitated from tobacco extracts (12 h salt‐treated or 48 h GmCSN5a‐coexpressed) using GFP‐Trap Magnetic Agarose (Chromotek GTMA‐20). To evaluate the enzyme active site, affinity‐purified GST‐GmRNF1a4Y (BeyoGold P2251) was used in ubiquitination reactions, omitting substrate proteins. GmRNF1a‐GFP complexes were isolated from tobacco proteins via GFP bead precipitation, and ubiquitination status was determined by sequential immunoblotting with anti‐GFP (Abcam ab290, 1:10,000), anti‐MYC (Proteintech 60003‐2‐Ig, 1:5,000), and anti‐Ub antibodies.

### 4.8 In Vivo Degradation Assay

Protein degradation assays were performed as described [44]. Agrobacterium suspensions carrying GmSIN1‐MYC were mixed with GmRNF1a‐GFP or GFP control at OD600‐adjusted ratios (9:9, 9:6, 9:3, 9:0) and co‐infiltrated into tobacco leaves. After 48 h co‐cultivation at 25 °C under 16‐h light, leaf discs were homogenized in RIPA buffer (50 mM Tris‐HCl pH 7.4, 150 mM NaCl, 1% Triton X‐100) supplemented with protease inhibitors. Protein extracts were immunoblotted with anti‐MYC (1:5,000), anti‐GFP (1:10,000), and anti‐GmACTIN (1:5,000) antibodies, using GmACTIN as the loading control. Parallel RNA samples were reverse‐transcribed with Oligo (dT)20 primers, and semi‐quantitative RT–PCR (28 cycles) was performed using gene‐specific primers (Extended Data 1), normalized to *GmACTIN* expression.

### 4.9 Cell‐Free Degradation Assay

Root proteins were extracted from 10‐day‐old W82 and gmsse1 mutants using the extraction buffer (50 mM Tris‐HCl pH 8.0, 10 mM EDTA, 0.5 M sucrose, 1 mM MgCl_2_, 5 mM DTT). Purified MBP‐GmSIN1 was incubated with root extracts at 25 °C in the presence of 1 mM ATP for specified durations (0–6 h). Degradation kinetics were monitored by anti‐MBP immunoblotting (Proteintech 15089‐1‐AP, 1:5,000), with GmACTIN (1:3,000) as the loading control. Proteasome involvement was validated by parallel reactions containing 50 µM MG132. For salt‐stress experiments, plants were pretreated with 150 mM NaCl before protein extraction.

### 4.10 Transcriptome Sequencing and Analysis

Transcriptome profiling was performed on 7‐day‐old root tissues from wild‐type W82, *GmRNF1a‐OE* overexpressors, *gmsin1 gmsin1h* double mutants, and their 150 mM NaCl‐treated counterparts. RNA‐seq libraries were prepared by Beijing Genomics Institute (BGI) using poly (A) selection and sequenced on Illumina platforms. Differentially expressed genes (DEGs) between transgenic lines and W82 under salt stress were identified using the Dr. TOM analysis system (BGI) with thresholds of Benjamini–Hochberg adjusted *q*‐value ≤ 0.05 and |fold change| ≥ 0.5.

### 4.11 Haplotype Analysis

Using the Williams 82 genome as a reference, a genomic region covering a 2.0‐kb promoter fragment and the full‐length coding sequence of GmSIN1, GmCSN5a, and GmRNF1a was extracted from resequencing data of 592 soybean accessions. High‐quality SNPs were used to construct haplotype networks with Haploview software.

### 4.12 Statistical Analysis

For comparisons between two groups, Student's t‐test was applied. For multiple comparisons, one‐way ANOVA followed by Tukey's or LSD post hoc test was used. All analyses were performed using GraphPad Prism or Minitab.

### 4.13 Accession Numbers

Sequence data from this study can be found in the Phytozome database (https://phytozome‐next.jgi.doe.gov/) under the following accession numbers: *GmSIN1* (*Glyma.13G279900*), *GmSIN1h* (*Glyma.12G221500*), *GmRbohB‐1* (*Glyma.10G152200*), *GmRbohB‐2* (G*lyma.20G236200*), *GmRNF1a* (*Glyma.15G268100*), *GmCSN5a* (*Glyma.06G076000*). Raw RNA‐seq data have been deposited in the Genome Sequence Archive at the National Genomics Data Center (PRJCA040201).

## Author Contributions

F.N.X. conceived the project. J.L.X., S.L., and F.N.X. designed the experiments. J.L.X. drafted the manuscript. J.L.X. performed most of the experiments. M.Q.S. performed haplotypes analyses. X.X.Y., R.M.Z., M.K.Z., G.H.B., L.L.W., M.R.L., W.J.Z., M.Z.W., and T.S.L. performed parts of the biochemical, phenotyping, and transformation experiments. X.J.Z. and W.Y.Z. performed transgenic soybean lines generation and cultivation. F.N.X., S.L., and Q.L. revised the manuscript.

## Conflicts of Interest

The authors declare no conflicts of interest.

## Supporting information




**Supporting File 1**: advs74216‐sup‐0001‐SuppMat.docx.

**Supporting File 2**: advs74216‐sup‐0002‐TableS1.xlsx.

## Data Availability

The data that support the findings of this study are available on request from the corresponding author.

## References

[1] X. Liang , J. Li , Y. Yang , C. Jiang , and Y. Guo , “Designing Salt Stress‐Resilient Crops: Current Progress and Future Challenges,” Journal of Integrative Plant Biology 66 (2024): 303–329, 10.1111/jipb.13599. 38108117

[2] U. Deinlein , A. B. Stephan , T. Horie , W. Luo , G. Xu , and J. I. Schroeder , “Plant Salt‐Tolerance Mechanisms,” Trends in Plant Science 19 (2014): 371–379, 10.1016/j.tplants.2014.02.001. 24630845 PMC4041829

[3] M. M. Julkowska and C. Testerink , “Tuning Plant Signaling and Growth to Survive Salt,” Trends in Plant Science 20 (2015): 586–594, 10.1016/j.tplants.2015.06.008. 26205171

[4] J. K. Zhu , “Cell Signaling under Salt, Water and Cold Stresses,” Current Opinion in Plant Biology 4 (2001): 401–406, 10.1016/S1369-5266(00)00192-8. 11597497

[5] E. van Zelm , Y. Zhang , and C. Testerink , “Salt Tolerance Mechanisms of Plants,” Annual Review of Plant Biology 71 (2020): 403–433, 10.1146/annurev-arplant-050718-100005. 32167791

[6] C. Zhao , H. Zhang , C. Song , J. K. Zhu , and S. Shabala , “Mechanisms of Plant Responses and Adaptation to Soil Salinity,” Innovation 1 (2020): 100017. 34557705 10.1016/j.xinn.2020.100017 PMC8454569

[7] T. Jin , Y. Sun , Z. Shan , et al., “Natural Variation in the Promoter of GsERD15B Affects Salt Tolerance in Soybean,” Plant Biotechnology Journal 19 (2021): 1155–1169, 10.1111/pbi.13536. 33368860 PMC8196659

[8] H. S. Leung , L. Y. Chan , C. H. Law , M. W. Li , and H. M. Lam , “Twenty Years of Mining Salt Tolerance Genes in Soybean,” Molecular Breeding 43 (2023): 45, 10.1007/s11032-023-01383-3. 37313223 PMC10248715

[9] Y. Li , H. Ye , T. D. Vuong , et al., “A Novel Natural Variation in the Promoter of GmCHX1 Regulates Conditional Gene Expression to Improve Salt Tolerance in Soybean,” Journal of Experimental Botany 75 (2024): 1051–1062, 10.1093/jxb/erad404. 37864556 PMC10837011

[10] Y. Wu , J. Yuan , L. Shen , et al., “A Phosphorylation‐Regulated NPF Transporter Determines Salt Tolerance by Mediating Chloride Uptake in Soybean Plants,” The EMBO Journal 44: 923–946, 10.1038/s44318-024-00357-1. PMC11790925 39753952

[11] W. Zhang , X. Liao , Y. Cui , et al., “A Cation Diffusion Facilitator, GmCDF1, Negatively Regulates Salt Tolerance in Soybean,” PLOS Genetics 15 (2019): 1007798, 10.1371/journal.pgen.1007798. PMC6336350 30615606

[12] C. Wang , X. Li , Y. Zhuang , et al., “A Novel miR160a—GmARF16 – GmMYC2 Module Determines Soybean Salt Tolerance and Adaptation,” New Phytologist 241 (2024): 2176–2192, 10.1111/nph.19503. 38135657

[13] Y. Liang , H. J. Liu , J. Yan , and F. Tian , “Natural Variation in Crops: Realized Understanding, Continuing Promise,” Annual Review of Plant Biology 72 (2021): 357–385, 10.1146/annurev-arplant-080720-090632. 33481630

[14] A. N. Olsen , H. A. Ernst , L. L. Leggio , and K. Skriver , “NAC Transcription Factors: Structurally Distinct, Functionally Diverse,” Trends in Plant Science 10 (2005): 79–87, 10.1016/j.tplants.2004.12.010. 15708345

[15] S. Puranik , P. P. Sahu , P. S. Srivastava , and M. Prasad , “NAC Proteins: Regulation and Role in Stress Tolerance,” Trends in Plant Science 17 (2012): 369–381, 10.1016/j.tplants.2012.02.004. 22445067

[16] J. Tao , F. Wu , H. Wen , et al., “RCD1 Promotes Salt Stress Tolerance in Arabidopsis by Repressing ANAC017 Activity,” International Journal of Molecular Sciences 24 (2023): 9793. 37372941 10.3390/ijms24129793 PMC10298584

[17] W. Yao , K. Zhao , Z. Cheng , X. Li , B. Zhou , and T. Jiang , “Transcriptome Analysis of Poplar under Salt Stress and Over‐Expression of Transcription Factor NAC57 Gene Confers Salt Tolerance in Transgenic Arabidopsis,” Frontiers in Plant Science 9 (2018): 1121, 10.3389/fpls.2018.01121. 30233602 PMC6131821

[18] L. Zheng , Y. Hu , T. Yang , et al., “A Root Cap‐Localized NAC Transcription Factor Controls Root Halotropic Response to Salt Stress in Arabidopsis,” Nature Communications 15 (2024): 2061, 10.1038/s41467-024-46482-7. PMC10917740 38448433

[19] Z. Xu , Gongbuzhaxi , C. Wang , F. Xue , H. Zhang , and W. Ji , “Wheat NAC Transcription Factor TaNAC29 Is Involved in Response to Salt Stress,” Plant Physiology and Biochemistry 96 (2015): 356–363, 10.1016/j.plaphy.2015.08.013. 26352804

[20] X. Zhang , Y. Long , X. Chen , et al., “A NAC Transcription Factor OsNAC3 Positively Regulates ABA Response and Salt Tolerance in Rice,” BMC Plant Biology 21 (2021): 546, 10.1186/s12870-021-03333-7. 34800972 PMC8605558

[21] Y. Hong , H. Zhang , L. Huang , D. Li , and F. Song , “Overexpression of a Stress‐Responsive NAC Transcription Factor Gene ONAC022 Improves Drought and Salt Tolerance in Rice,” Frontiers in Plant Science 7 (2016): 4, 10.3389/fpls.2016.00004. 26834774 PMC4722120

[22] Y. J. Hao , W. Wei , Q. X. Song , et al., “Soybean NAC Transcription Factors Promote Abiotic Stress Tolerance and Lateral Root Formation in Transgenic Plants,” The Plant Journal 68 (2011): 302–313, 10.1111/j.1365-313X.2011.04687.x. 21707801

[23] X. L. T. Hoang , N. N. Chuong , T. T. K. Hoa , et al., “The Drought‐Mediated Soybean GmNAC085 Functions as a Positive Regulator of Plant Response to Salinity,” International Journal of Molecular Sciences 22 (2021): 8986. 34445699 10.3390/ijms22168986 PMC8396556

[24] M. Li , R. Chen , Q. Jiang , X. Sun , H. Zhang , and Z. Hu , “GmNAC06, a NAC Domain Transcription Factor Enhances Salt Stress Tolerance in Soybean,” Plant Molecular Biology 105 (2021): 333–345, 10.1007/s11103-020-01091-y. 33155154 PMC7858558

[25] S. Li , N. Wang , D. Ji , et al., “A GmSIN1/GmNCED3s/GmRbohBs Feed‐Forward Loop Acts as a Signal Amplifier That Regulates Root Growth in Soybean Exposed to Salt Stress,” The Plant Cell 31 (2019): 2107–2130, 10.1105/tpc.18.00662. 31227558 PMC6751118

[26] X. Yang , M. Y. Kim , J. Ha , and S. H. Lee , “Overexpression of the Soybean NAC Gene GmNAC109 Increases Lateral Root Formation and Abiotic Stress Tolerance in Transgenic Arabidopsis Plants,” Frontiers in Plant Science 10 (2019): 1036, 10.3389/fpls.2019.01036. 31475026 PMC6707213

[27] W. Zhang , W. Zhi , H. Qiao , et al., “H_2_O_2_‐Dependent Oxidation of the Transcription Factor GmNTL1 Promotes Salt Tolerance in Soybean,” The Plant Cell 36 (2023): 112–135, 10.1093/plcell/koad250. 37770034 PMC10734621

[28] L. D. Vu , K. Gevaert , and I. De Smet , “Protein Language: Post‐Translational Modifications Talking to each Other,” Trends in Plant Science 23 (2018): 1068–1080, 10.1016/j.tplants.2018.09.004. 30279071

[29] K. N. Swatek and D. Komander , “Ubiquitin Modifications,” Cell Research 26 (2016): 399–422, 10.1038/cr.2016.39. 27012465 PMC4822133

[30] R. Al‐Saharin , H. Hellmann , and S. Mooney , “Plant E3 Ligases and Their Role in Abiotic Stress Response,” Cells 11 (2022): 890. 35269512 10.3390/cells11050890 PMC8909703

[31] Z. Ban and M. Estelle , “CUL3 E3 Ligases in Plant Development and Environmental Response,” Nature Plants 7 (2021): 6–16, 10.1038/s41477-020-00833-6. 33452490 PMC8932378

[32] Y. Meng , Q. Lv , L. Li , et al., “E3 ubiquitin Ligase TaSDIR1‐4A Activates Membrane‐Bound Transcription Factor TaWRKY29 to Positively Regulate Drought Resistance,” Plant Biotechnology Journal 22 (2024): 987–1000, 10.1111/pbi.14240. 38018512 PMC10955488

[33] H. Yi , H. Shi , W. Mao , et al., “E3 ubiquitin Ligase IPI1 Controls Rice Immunity and Flowering via both E3 Ligase‐Dependent and ‐Independent Pathways,” Developmental Cell 59 (2024): 2719–2730, 10.1016/j.devcel.2024.06.014. 39025062

[34] T. Wu , Y. Wang , J. Jin , et al., “Soybean RING ‐Type E 3 Ligase G M CHYR 16 Ubiquitinates the G M ERF 71 Transcription Factor for Degradation to Negatively Regulate Bicarbonate Stress Tolerance,” New Phytologist 246: 1128–1146, 10.1111/nph.70041. 40079647

[35] R. Luo , K. Yang , and W. Xiao , “Plant Deubiquitinases: from Structure and Activity to Biological Functions,” Plant Cell Reports 42 (2023): 469–486, 10.1007/s00299-022-02962-y. 36567335

[36] E. Isono and M. K. Nagel , “Deubiquitylating Enzymes and Their Emerging Role in Plant Biology,” Frontiers in Plant Science 5 (2014): 56, 10.3389/fpls.2014.00056. 24600466 PMC3928566

[37] W. Dubiel , S. Chaithongyot , D. Dubiel , and M. Naumann , “The COP9 Signalosome: a Multi‐DUB Complex,” Biomolecules 10 (2020): 1082. 32708147 10.3390/biom10071082 PMC7407660

[38] C. Schwechheimer and E. Isono , “The COP9 Signalosome and Its Role in Plant Development,” European Journal of Cell Biology 89 (2010): 157–162, 10.1016/j.ejcb.2009.11.021. 20036030

[39] X. I. Ambroggio , D. C. Rees , and R. J. Deshaies , “JAMM: a Metalloprotease‐Like Zinc Site in the Proteasome and Signalosome,” PLoS Biology 2 (2004): 2. 10.1371/journal.pbio.0020002 PMC300881 14737182

[40] N. Qin , D. Xu , J. Li , and X. W. Deng , “COP9 signalosome: Discovery, Conservation, Activity, and Function,” Journal of Integrative Plant Biology 62 (2020): 90–103, 10.1111/jipb.12903. 31894894

[41] C. Schwechheimer , “NEDD8 — its Role in the Regulation of Cullin‐RING Ligases,” Current Opinion in Plant Biology 45 (2018): 112–119, 10.1016/j.pbi.2018.05.017. 29909289

[42] C. Feng , M. A. Hussain , Y. Zhao , et al., “GmAKT1 ‐mediated K + Absorption Positively Modulates Soybean Salt Tolerance by GmCBL9 ‐ GmCIPK6 Complex,” Plant Biotechnology Journal 23 (2025): 2276–2289, 10.1111/pbi.70042. 40112140 PMC12120911

[43] X. Ni , Y. Wang , L. Dai , et al., “The Transcription Factor GmbZIP131 Enhances Soybean Salt Tolerance by Regulating Flavonoid Biosynthesis,” Plant Physiology 197 (2025): kiaf092. 40073410 10.1093/plphys/kiaf092

[44] B. Li , J. Peng , Y. Wu , et al., “Identification of an Important QTL for Seed Oil Content in Soybean,” Molecular Breeding 43 (2023): 43, 10.1007/s11032-023-01384-2. 37313220 PMC10248617

[45] Z. Yang , Y. Chi , Y. Cui , et al., “Ectopic Expression of GmRNF1a Encoding a Soybean E3 Ubiquitin Ligase Affects Arabidopsis Silique Development and Dehiscence,” Planta 255 (2022): 55, 10.1007/s00425-022-03833-2. 35106662

[46] J. Wang , C. Zhang , H. Li , et al., “OsJAB1 Positively Regulates Ascorbate Biosynthesis and Negatively Regulates Salt Tolerance due to Inhibiting Early‐Stage Salt‐Induced ROS Accumulation in Rice,” Plants 12 (2023): 3859. 38005759 10.3390/plants12223859 PMC10675544

[47] M. J. Skelly , “The Emerging Roles of Deubiquitinases in Plant Proteostasis,” Essays in Biochemistry 66 (2022): 147–154. 35678302 10.1042/EBC20210060 PMC9400064

[48] A. K. Singh , S. Dhanapal , A. Finkelshtein , and D. A. Chamovitz , “CSN5A Subunit of COP9 Signalosome Is Required for Resetting Transcriptional Stress Memory after Recurrent Heat Stress in Arabidopsis,” Biomolecules 11 (2021): 668. 33946149 10.3390/biom11050668 PMC8146153

