# Supplementary material for: A Novel GmSIN1‐GmRNF1a‐GmCSN5a Module Determines Soybean Salt Tolerance and Yield Under Saline Soil Conditions
Source: Adv Sci (Weinh). 2026 Feb 15;13(21):e74216. doi: 10.1002/advs.74216 (PMC13073264; doi:10.1002/advs.74216)
Supplement: Supplementary file 1 — Supporting File 1: advs74216‐sup‐0001‐SuppMat.docx. [file ADVS-13-e74216-s002.docx]

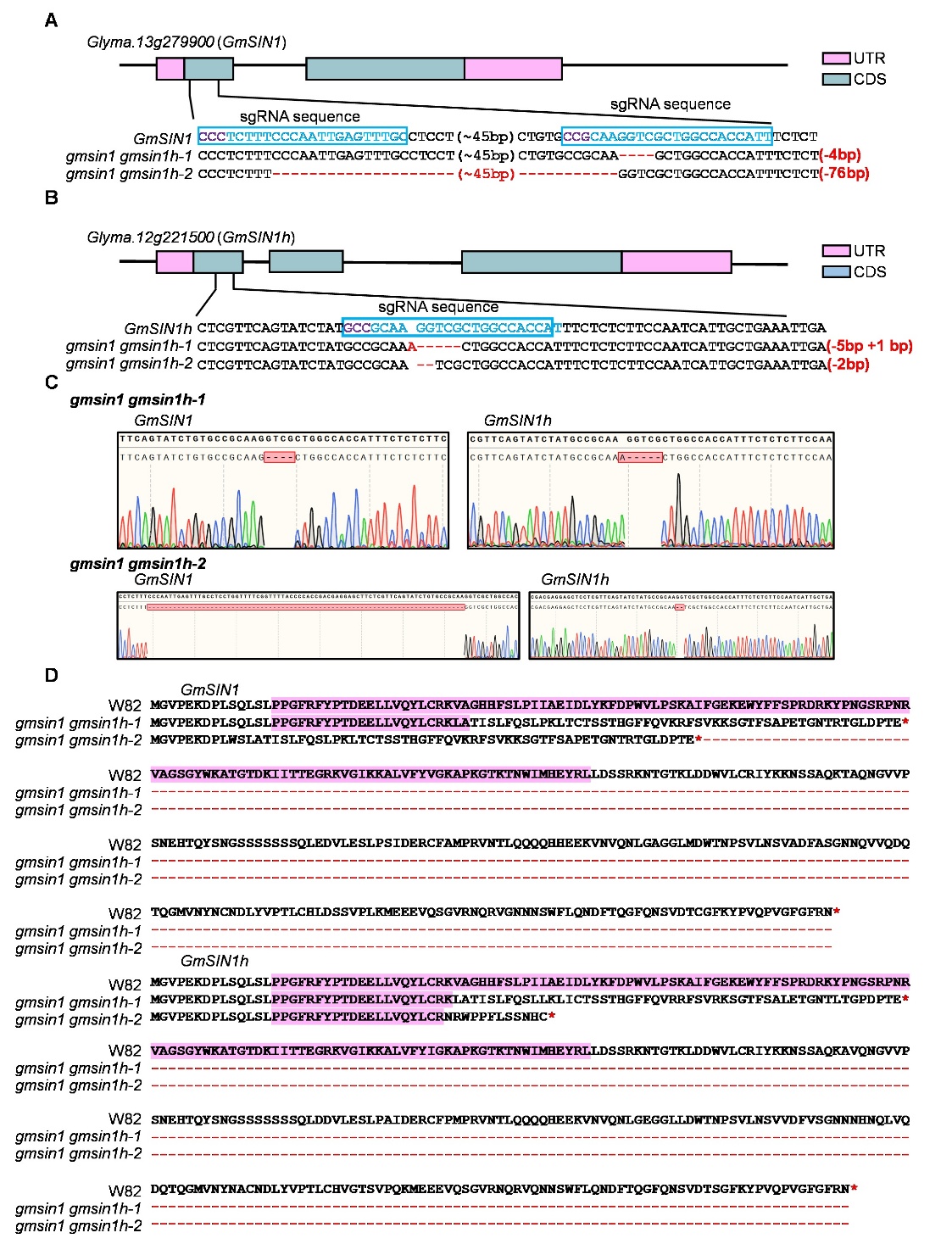


**Figure S1.** Detection of transgenic soybean plants with GmSIN1 edited via CRISPR-Cas9. A–B), Schematic diagrams of the *GmSIN1* and its homolog *GmSIN1h* gene structures, along with their editing status in the generated mutants. Gray regions represent UTRs, and blue regions denote exons. Purple-highlighted bases indicate PAM sites; teal boxes mark sgRNA sequences; red lines represent deleted nucleotides, with the deletion lengths specified by red numbers in parentheses. C) The sequences of *GmSIN1* and *GmSIN1h* in *gmsin1 gmsin1h-1* and *gmsin1 gmsin1h-2* were generated using CRISPR-Cas9-mediated editing. D) The protein sequences encoded by the *gmsin1 gmsin1h-1* and *gmsin1 gmsin1h-2* mutant alleles were altered due to CRISPR-Cas9 editing.


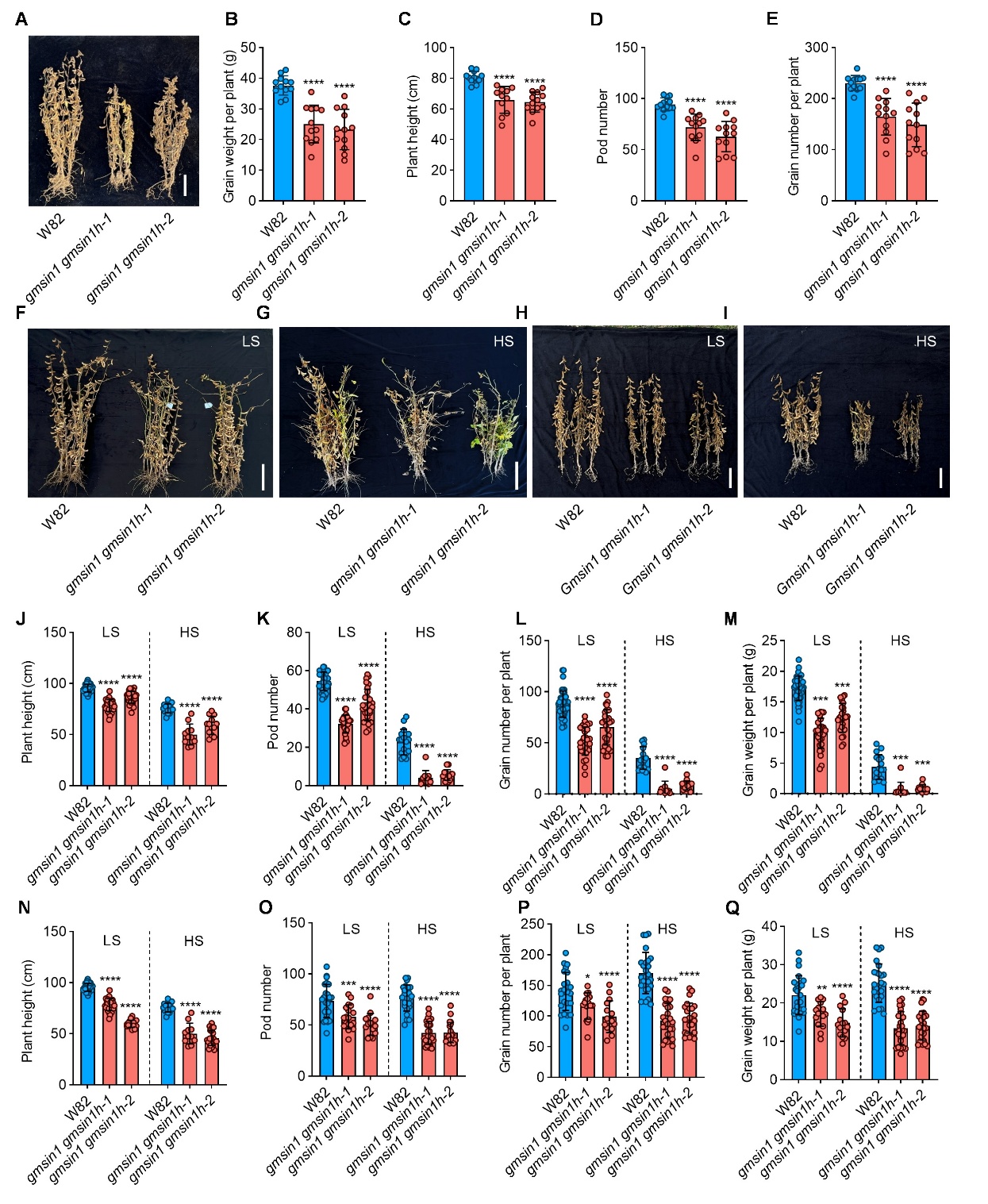


**Figure S2.** Loss of *GmSIN1* function reduces salt tolerance in soybean. A) Representative plant growth phenotypes of the wild-type (WT) and *gmsin1* mutants under control conditions. Scale bar = 13 cm. B–E) Quantitative analysis of plant height (B), pod number per plant (C), seed number per plant (D), and seed weight per plant (E) under control conditions, corresponding to the plants shown in (A). F–G) Plant growth phenotypes under low- and high-salinity stress, respectively, from the 2024 trial. Scale bar = 13 cm. H–I) Plant growth phenotypes under low- and high-salinity stress, respectively, from the 2025 trial. Scale bar = 13 cm. J–M) Quantitative data for plant height (J), pod number (K), seed number (L), and seed weight (M) per plant from the 2024 salinity trials [corresponding to (F) and (G)]. N–Q) Quantitative data for plant height (N), pod number (O), seed number (P), and seed weight (Q) per plant from the 2025 salinity trials [corresponding to (H) and (I)]. All the data points are shown on the plots. Data in (B)–(E) are presented as mean ± SD (n ≥ 12, 3 different sites). Data in J–Q) are presented as mean ± SD (n ≥ 15, 3 different sites). These data were analyzed by one-way ANOVA followed by Tukey’s test (two-sided), asterisks indicate significant differences (**P* < 0.05; ***P* < 0.01; ****P* < 0.001; *****P* < 0.0001).


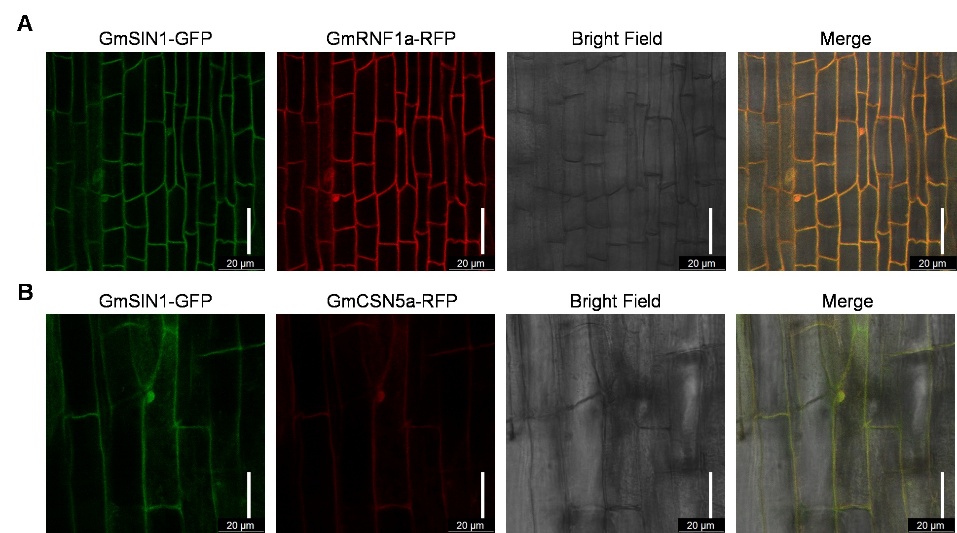


**Figure S3.** Subcellular co-localization of *GmSIN1* with *GmRNF1a* and *GmCSN5a*. A) Co-localization of GmSIN1-GFP with GmRNF1a-RFP in plant cells. B) Co-localization of GmSIN1-GFP with GmCSN5a-RFP in plant cells. Image presentation: For each experiment (a, b), four representative panels are shown (from left to right): GFP fluorescence channel (GmSIN1-GFP), RFP fluorescence channel (GmRNF1a-RFP or GmCSN5a-RFP), bright-field image, and merged channel image. Scale bar = 20 μm.


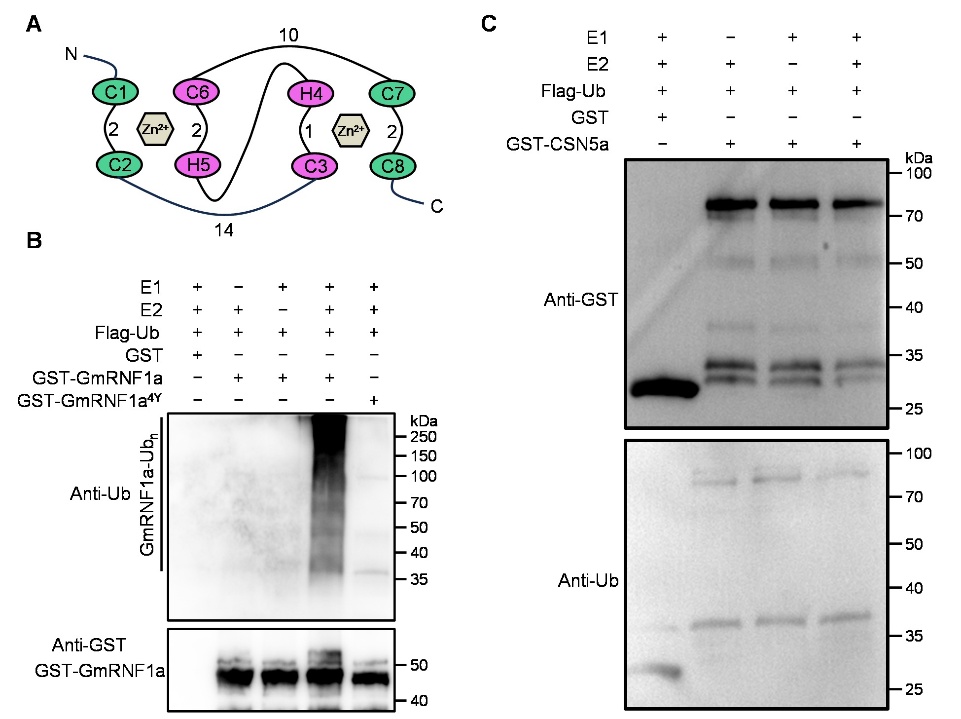


**Figure S4.** Structural features and E3 ligase activity validation of GmRNF1a. A) Key residues in the zinc finger domain of GmRNF1a. Cyan: four cysteine (C) ligands; red: four amino acids forming the E3 ubiquitin ligase active center; Zn²⁺ indicates the predicted metal-binding site. B) *In vitro* ubiquitination assay demonstrating the E3 ligase activity of GmRNF1a. No polyubiquitination signal was observed in reactions lacking E1 or E2. In the presence of E1 and E2, GST-GmRNF1a (but not GST alone) produced a characteristic polyubiquitination smear, which was abolished by mutating the active-site residues. C) GmCSN5a lacks E3 ligase activity. Under identical *in vitro* ubiquitination conditions (E1, E2, Ub present), no polyubiquitination was detected for GmCSN5a.


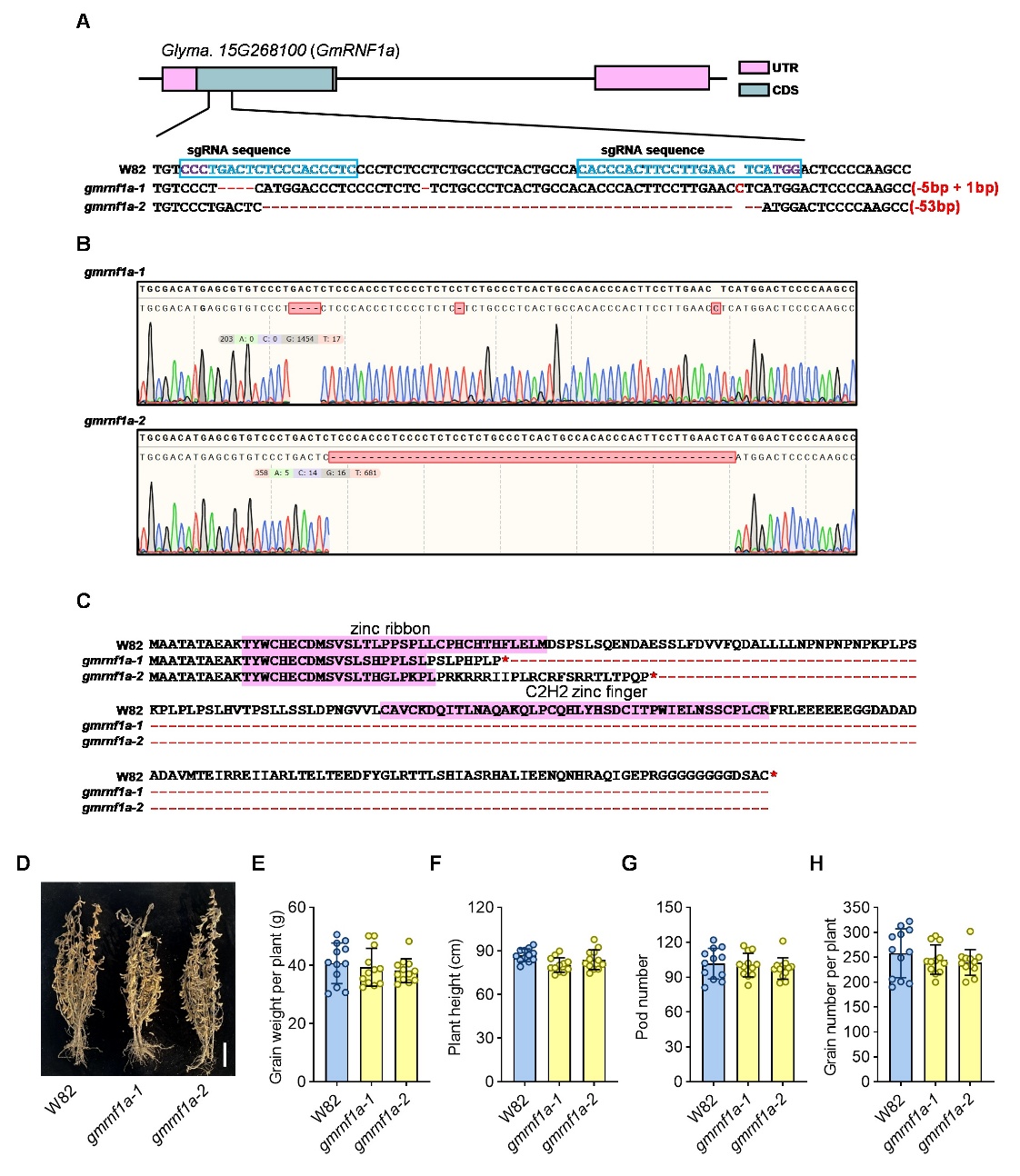


**Figure S5.** Generation and phenotypic characterization of *gmrnf1a* CRISPR-Cas9 mutants. A) Schematic of the GmRNF1a genomic structure (top) and CRISPR-Cas9 editing outcomes in mutants (bottom). Gray boxes: UTRs; blue boxes: exons. Purple bases: PAM sites; teal boxes: sgRNA target sequences; red lines: deleted nucleotides; red numbers in parentheses: deletion length. B) Sequencing chromatograms confirming the edited DNA sequences in *gmrnf1a-1* and *gmrnf1a-2* mutants. C) Predicted protein sequences translated from the mutant alleles, showing frameshift-induced alterations. D) Representative mature plant phenotypes of wild-type (W82) and *gmrnf1a* mutants under normal growth conditions. Scale bar = 13 cm. E–H) Quantitative analysis of plant height (E), pod number per plant (F), seed number per plant (G), and seed weight per plant (H) corresponding to the plants shown in D. All the data points are shown on the plots. Data in E–H are presented as mean ± SD (n ≥ 12, 3 different sites).


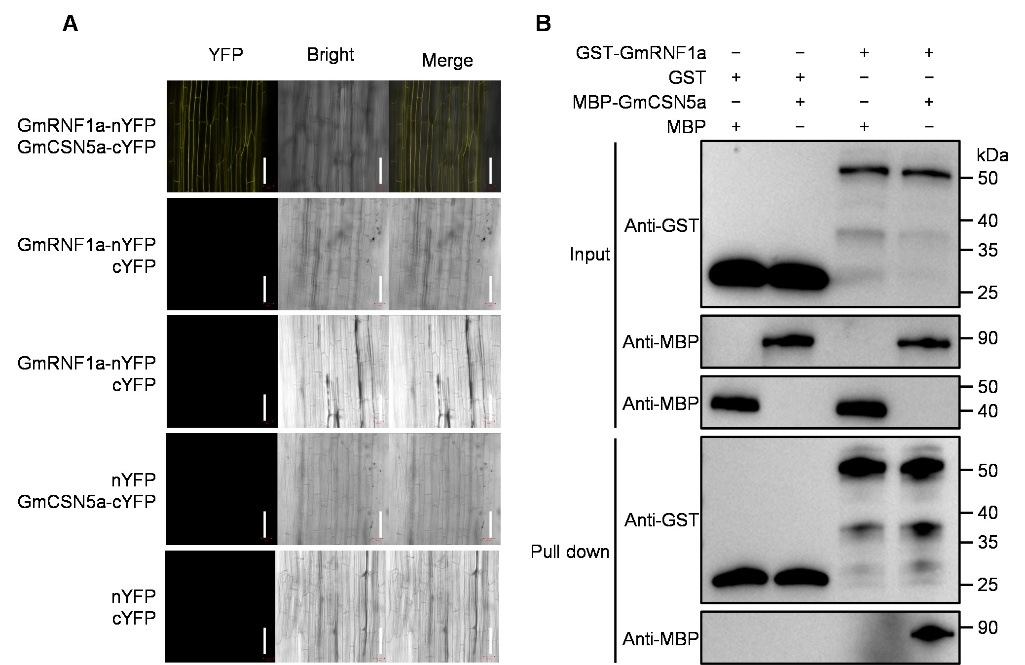


**Figure S6.** Interaction between GmRNF1a and GmCSN5a demonstrated by pull-down and BiFC assays. A) Bimolecular fluorescence complementation (BiFC) assay showing the interaction between GmRNF1a and GmCSN5a in transgenic soybean hairy roots. Scale bar = 20 μm. B) *In vitro* pull-down assay confirms the direct interaction. MBP-tagged GmCSN5a was pulled down by GST-tagged GmRNF1a and detected using an anti-MBP antibody. Input levels of GST-GmRNF1a and MBP-GmCSN5a were verified with anti-GST and anti-MBP antibodies, respectively. MBP and GST tags alone served as negative controls.


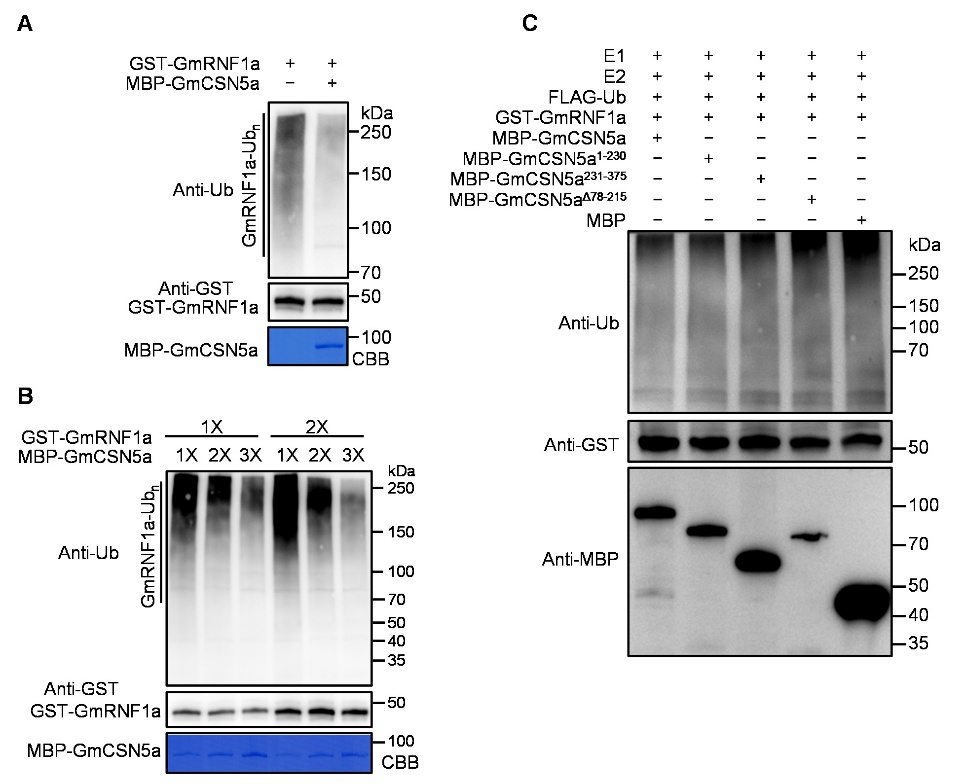


**Figure S7.** GmCSN5a directly inhibits the E3 ligase activity of GmRNF1a via its MPN domain. A) *In vitro* ubiquitination assay showing that GmCSN5a suppresses GmRNF1a-mediated polyubiquitination. Polyubiquitination of GmRNF1a was detected using an anti-ubiquitin (Ub) antibody. GST-GmRNF1a levels were monitored with an anti-GST antibody, and the presence of MBP-GmCSN5a was confirmed by Coomassie Brilliant Blue (CBB) staining. B) Dose-dependent inhibition of GmRNF1a E3 ligase activity by GmCSN5a. The polyubiquitination signal decreased with increasing concentrations of MBP-GmCSN5a. Assay conditions and detection methods were identical to those in (A). C) GmCSN5a directly inhibits GmRNF1a-mediated ubiquitination via its MPN domain. *In vitro* ubiquitination assays were performed using purified full-length GmCSN5a or its truncated variants. Only the construct containing the MPN domain retained the ability to inhibit GmRNF1a E3 activity. MBP alone served as a negative control. Polyubiquitination was detected with an anti-Ub antibody.


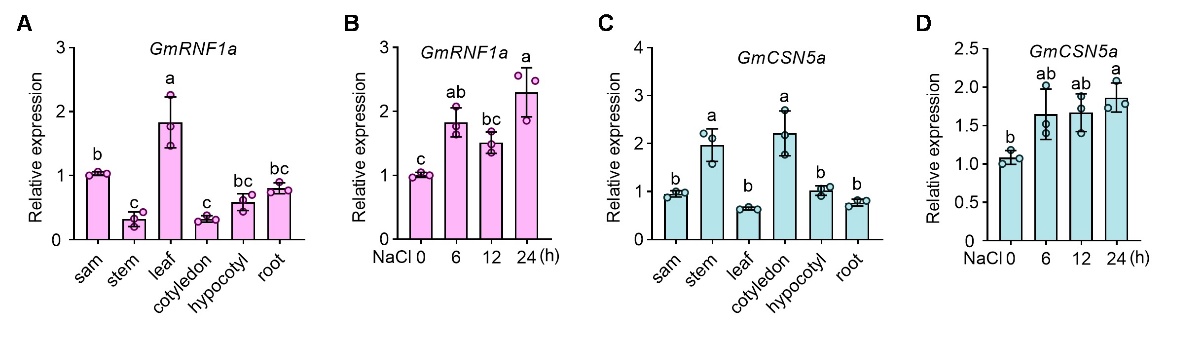


**Figure S8.** Tissue-specific and salt stress-induced expression patterns of GmRNF1a and GmCSN5a. A, C) Tissue-specific expression profiles of GmRNF1a (A) and GmCSN5a (C) in shoot apical meristem (SAM), stem, leaf, cotyledon, hypocotyl, and root. Data are presented as mean ± SD (n = 3 biological replicates). B, D) Expression dynamics of GmRNF1a (B) and GmCSN5a (D) in response to salt stress (150 mM NaCl) over a 24-hour time course. All the data points are shown on the plots. Data in (A)–(D) are presented as mean ± SD (n = 3 biological replicates). These data were analyzed by one-way ANOVA followed by Tukey’s test (two-sided), different letters indicate significant differences (*P* < 0.05).


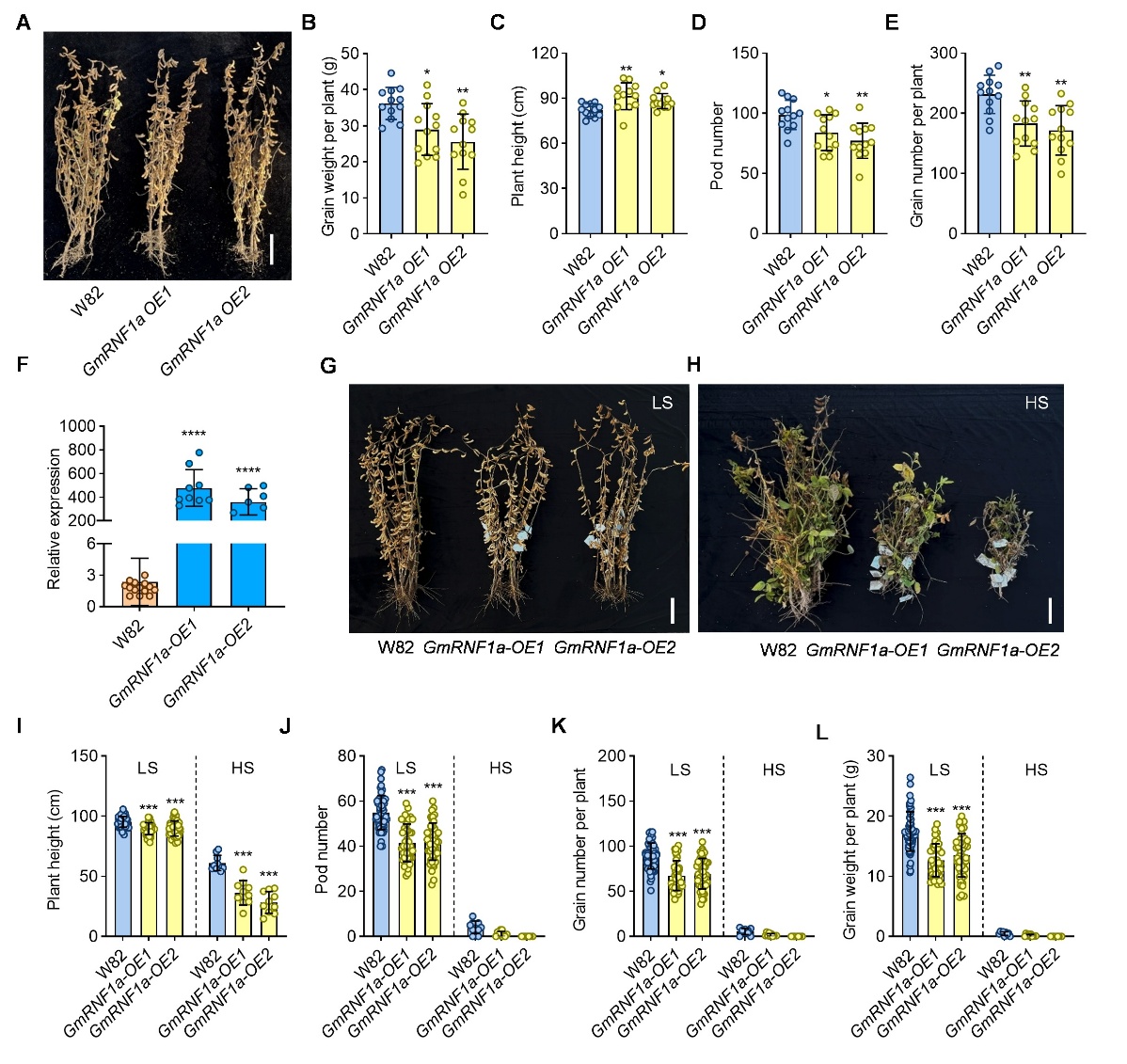


**Figure S9.** Phenotypic analysis of GmRNF1a-overexpressing (OE) plants under normal and salt-stress conditions. A) Phenotype of wild-type (W82) and *GmRNF1a-OE* plants under normal growth conditions. Scale bar = 13 cm. B–E) Quantitative analysis of seed weight per plant (B), plant height (C), pod number per plant (D), and grain number per plant (E), respectively. F) RT‑qPCR validation of *GmRNF1a* overexpression in transgenic lines. G–H) Representative plant images under low- and high-salinity conditions, respectively. Scale bar = 13 cm. I–L) Corresponding quantitative analysis of plant height (I), pod number (J), seed number (K), and seed weight per plant (L) under salt stress. All the data points are shown on the plots. Data in F are presented as mean ± SD (n = 7 biological replicates). Data in (B)–(E) and (I)–(L) are presented as mean ± SD (n ≥ 12, 3 different sites). These data were analyzed by one-way ANOVA followed by Fisher’s LSD test (two-sided), asterisks indicate significant differences (**P* < 0.05; ***P* < 0.01; ****P* < 0.001).


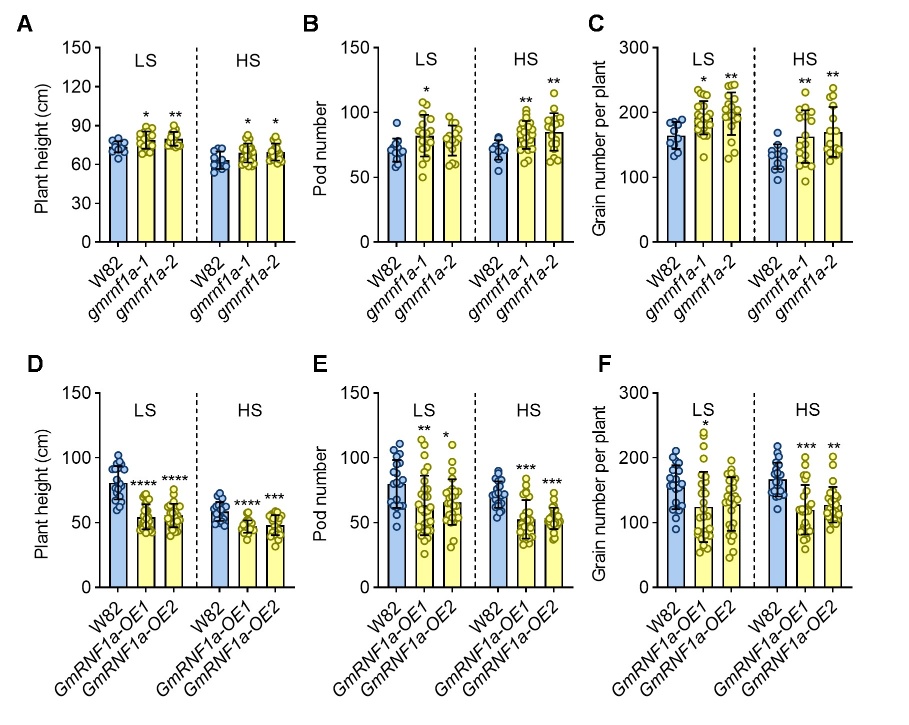


**Figure S10****.** Supplementary yield-related trait data for GmRNF1a transgenic lines under salinity (related to Figure 5). Quantitative analysis of key agronomic traits—plant height, pods per plant, seeds per plant, and grain weight per plant—in *gmrnf1a* mutants (A)–(C) and *GmRNF1a-OE* lines (D)–(F) under two different salinity levels during the 2023 and 2024 growing seasons, respectively. All the data points are shown on the plots. Data in A–F) are presented as mean ± SD (n ≥ 15, 3 different sites). These data were analyzed by one-way ANOVA followed by Fisher’s LSD test (two-sided), asterisks indicate significant differences (**P* < 0.05; ***P* < 0.01; ****P* < 0.001; *****P* < 0.0001).


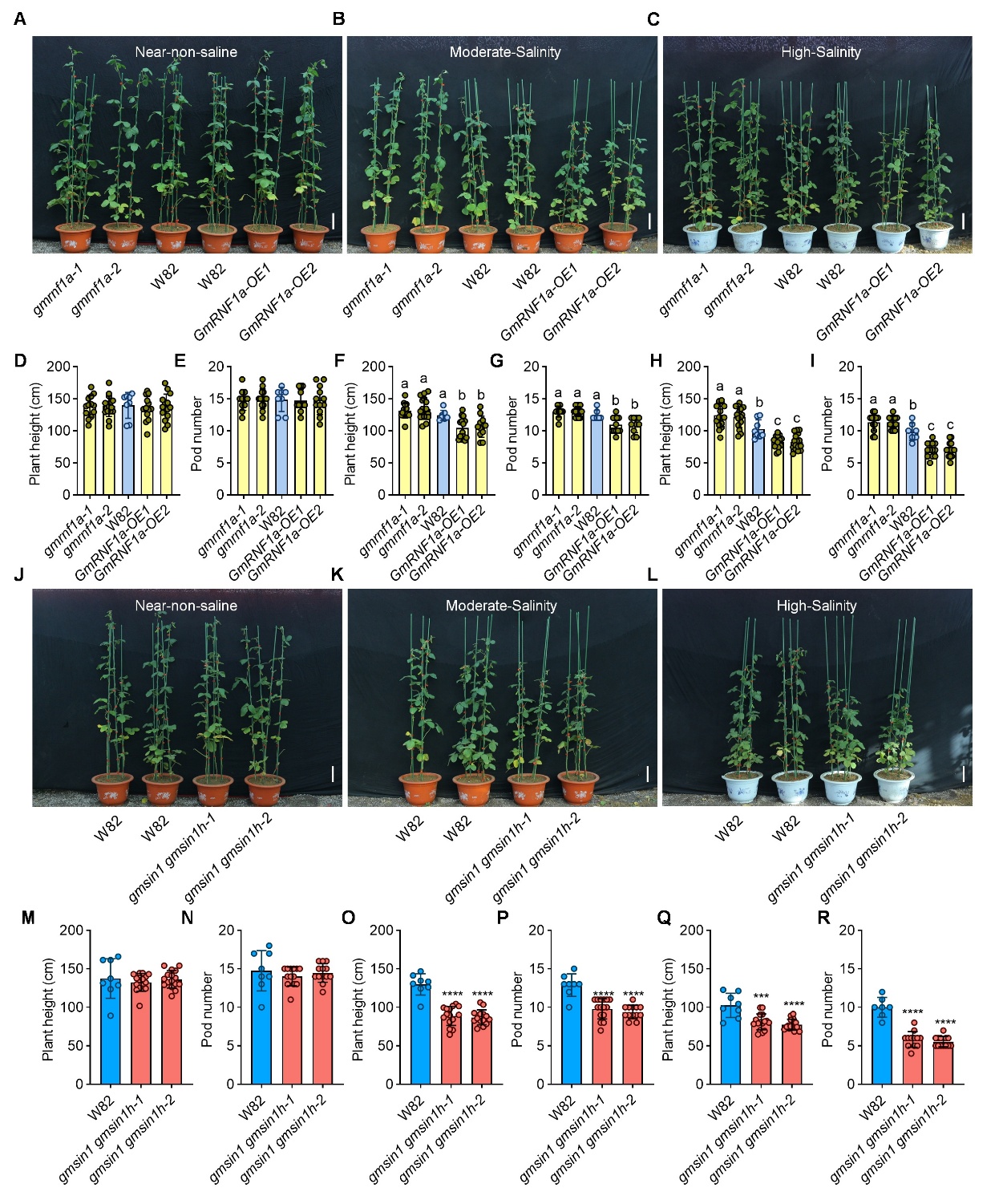


**Figure S11.** Salt stress phenotypes of soybean mutant lines under varying salinity levels. A–C) Phenotype of *GmRNF1a-OE* and *gmrnf1a* plants under three salinity levels (non-, moderate, and high salinity). Scale bar = 20 cm. (D), (F), (H) Plant height of *GmRNF1a-OE* and *gmrnf1a* plants under the three salinity conditions, compared to the wild type (W82). (E), (G), (I) Pod number per plant of *GmRNF1a-OE* and *gmrnf1a* plants under the three salinity conditions, compared to the wild type (W82). J–L) Phenotype of *gmsin1 gmsin1h* plants under the three salinity levels. (M), (O), (Q) Plant height of *gmsin1 gmsin1h* plants under the three salinity conditions, compared to the wild type (W82). (N), (P), (R) Pod number per plant of *gmsin1 gmsin1h* plants under the three salinity conditions, compared to the wild type (W82). All the data points are shown on the plots. Data in D–I) are presented as mean ± SD (n ≥ 8 biological replicates). These data were analyzed by one-way ANOVA followed by Fisher’s LSD test (two-sided), different letters indicate significant differences (*P* < 0.05). Data in M–R) are presented as mean ± SD (n ≥ 8 biological replicates). These data were analyzed by one-way ANOVA followed by Fisher’s LSD test (two-sided), asterisks indicate significant differences (****P* < 0.001; *****P* < 0.0001).


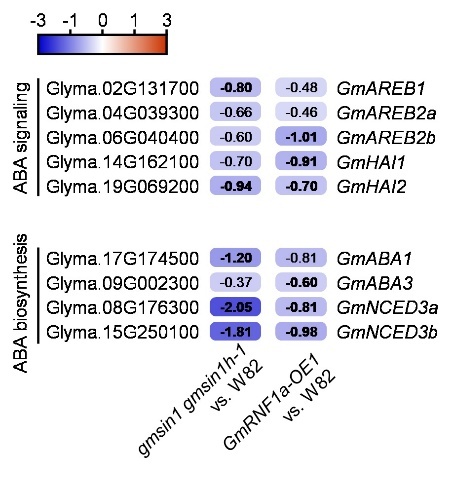


**Figure S12.** Expression patterns of ABA biosynthesis and signaling genes under salt stress. Differential expression of ABA pathway genes was assessed using RNA‑seq data from comparisons between *gmsin1 gmsin1h* mutants versus wild‑type (WT) and *GmRNF1a-OE* lines versus WT under salt stress. The color scale represents log2 fold‑change values (red: maximum upregulation, 3; blue: maximum downregulation, –3). Rounded rectangles display the specific fold‑change value for each gene.


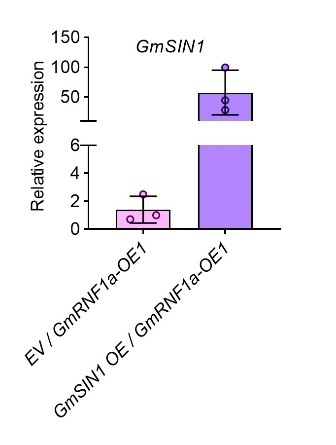


**Figure S13.** Expression analysis of GmSIN1 in transgenic soybean hairy roots. Quantitative analysis of *GmSIN1* expression in empty vector (EV) control and *GmSIN1-OE* transgenic hairy roots generated in the *GmRNF1a-OE* background. All the data points are shown on the plots. Data are presented as mean ± SD (n = 3 biological replicates).


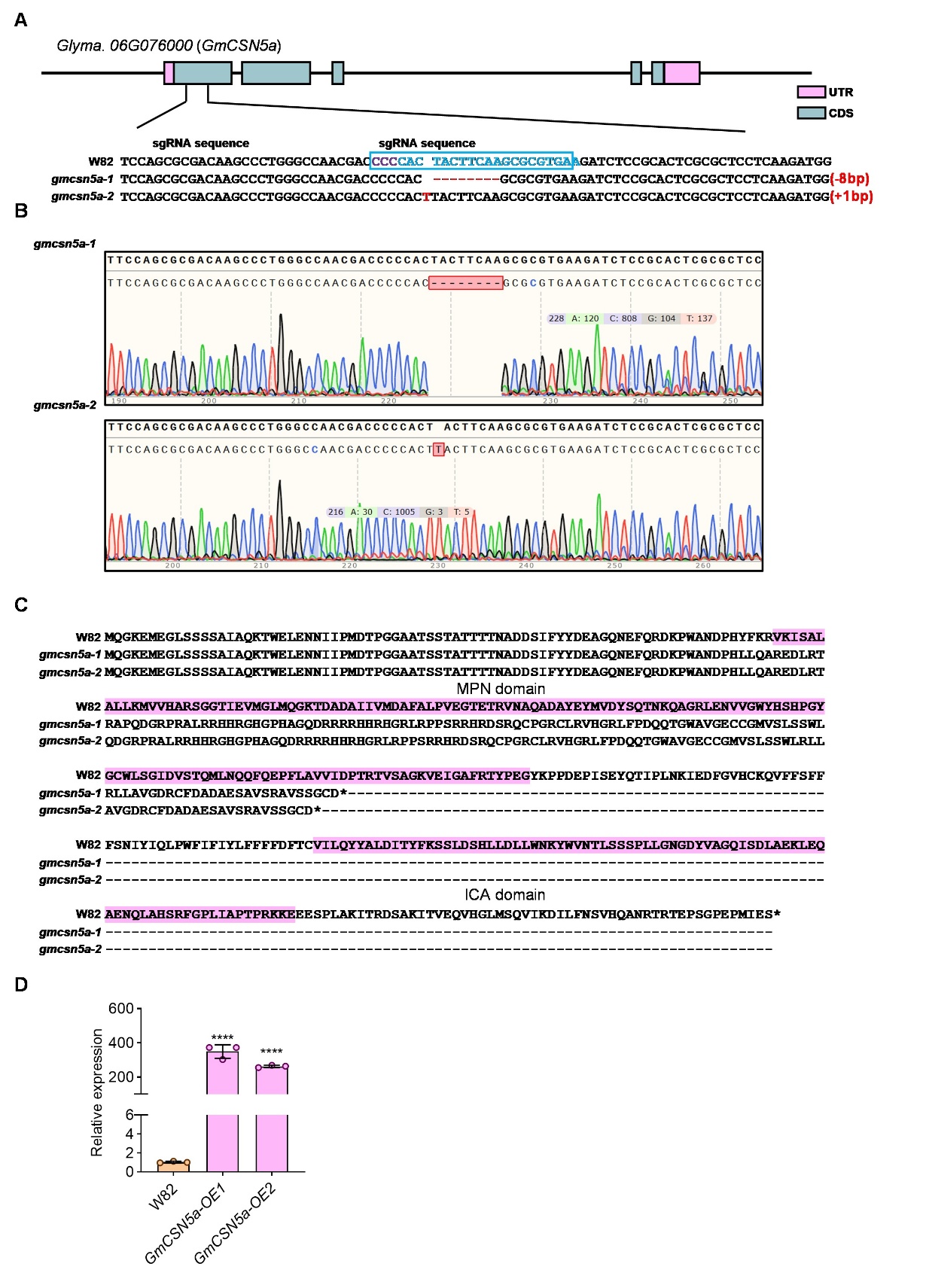


**Figure S14.** Generation and molecular characterization of *GmCSN5a* mutant and overexpression lines in soybean. A) Schematic of the *GmCSN5a* genomic locus (top) and *CRISPR‑Cas9* editing outcomes in mutant alleles (bottom). Gray and blue boxes represent UTRs and exons, respectively. The sgRNA sequence is highlighted in teal, and the PAM site is shown in purple. Red lines indicate deleted nucleotides, with deletion lengths noted in parentheses. B) DNA sequences of the edited regions in the *gmcsn5a*‑1 and *gmcsn5a*‑2 mutant lines. C) Corresponding protein sequences encoded by the wild‑type and mutant *gmcsn5a* alleles, showing alterations resulting from frameshift mutations. D) Relative expression levels of *GmCSN5a* in wild‑type (Shanda No.5) and *GmCSN5a‑OE* transgenic lines. All the data points are shown on the plots. Data are presented as mean ± SD (n = 3 biological replicates). These data were analyzed by one-way ANOVA followed by Fisher’s LSD test (two-sided), asterisks indicate significant differences (*****P* < 0.0001).


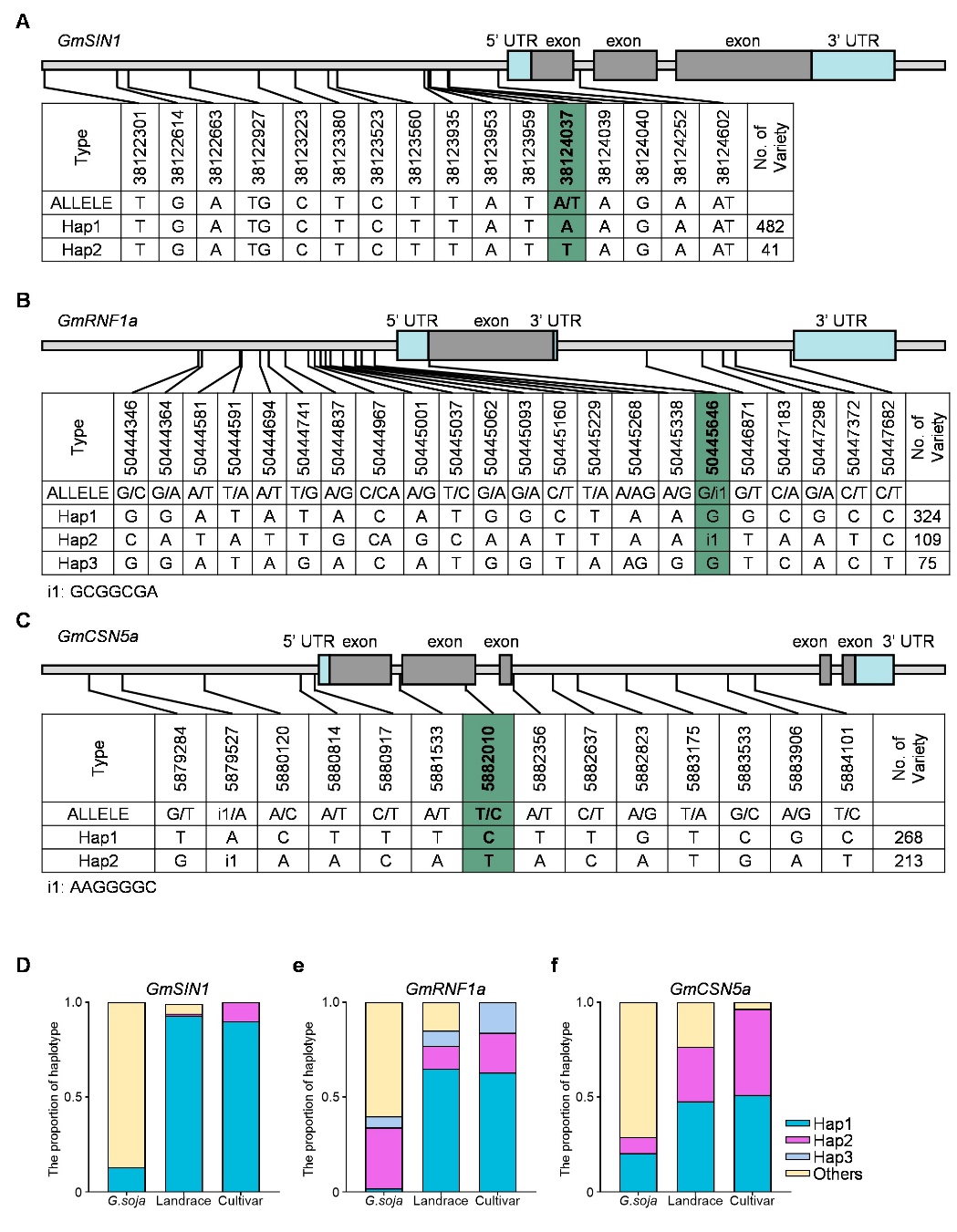


**Figure S15.** Haplotype analysis of *GmSIN1*, *GmRNF1a*, and *GmCSN5a* in diverse soybean accessions. A) Summary of natural haplotypes identified for GmSIN1. Top: schematic of the GmSIN1 gene structure, showing the promoter region (2,000 bp upstream of ATG), UTRs (sky blue), and exons (gray). Bottom: table listing variation sites and types for five major haplotypes (Hap1–Hap5), with the number of accessions per haplotype indicated in the last column. Nucleotide changes specific to Hap1 and Hap2 are highlighted in green. B–C) Haplotype summaries for GmRNF1a (B) and GmCSN5a (C), presented in the same format as in (A). D–F) Geographic‑/group‑based distribution of the predominant haplotypes of *GmSIN1* (D), *GmRNF1a* (E), and *GmCSN5a* (F) across wild soybean, landrace, and improved cultivar populations.


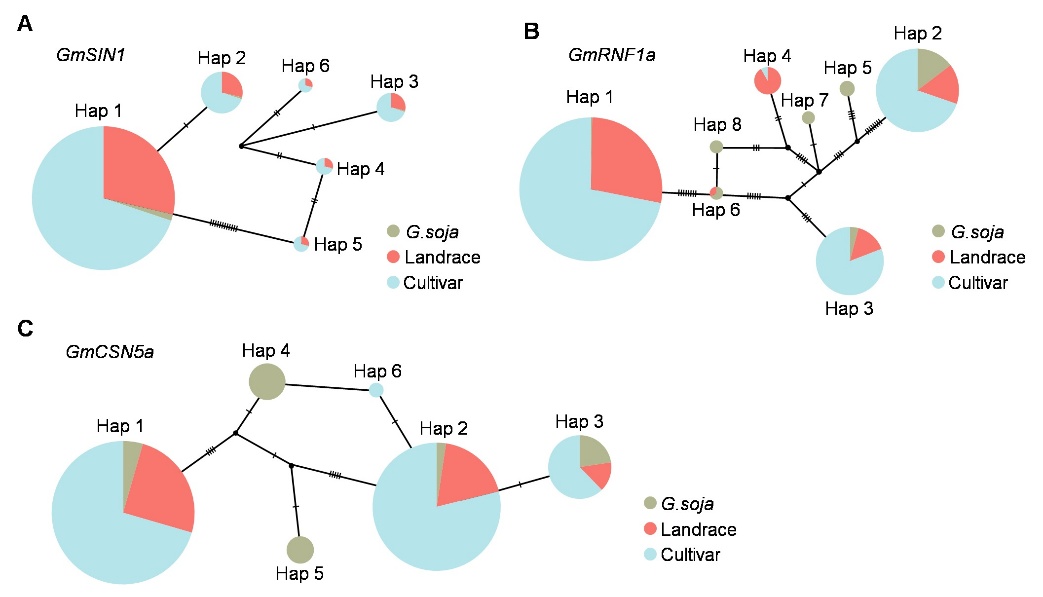


**Figure S16.** Haplotype network analysis of GmSIN1, GmRNF1a, and GmCSN5a in soybean populations. A–C) Median‑joining haplotype networks constructed for GmSIN1 (A), GmRNF1a (B), and GmCSN5a (C). Each circle represents a distinct haplotype; its size is proportional to the number of accessions carrying that haplotype. Colors denote the three soybean groups: olive for wild soybean (*G. soja*), rose red for landraces, and sky blue for improved cultivars. Connecting lines between circles indicate mutational steps between haplotypes.


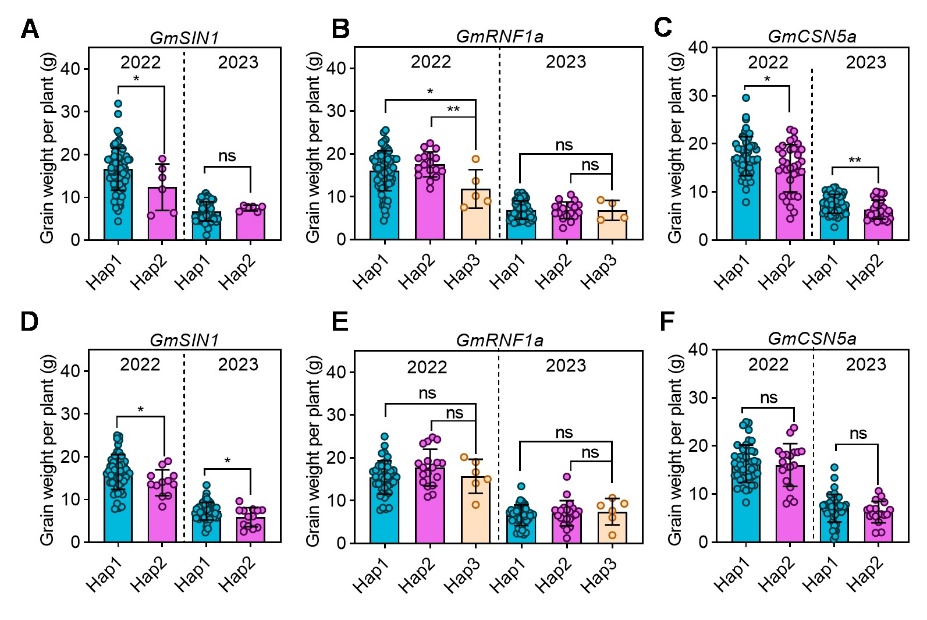


**Figure S17**. Haplotype-trait associations for grain weight in two major soybean ecotypes under salinity. A–C) Analysis of haplotype effects on seed weight per plant for *GmSIN1* (A), *GmRNF1a* (B), and *GmCSN5a* (C) was performed separately in the Northern Spring Soybean ecotype. D–F Analysis of haplotype effects on seed weight per plant for *GmSIN1* (D), *GmRNF1a* (E), and *GmCSN5a* (F) was performed separately in the Huang‑Huai‑Hai Summer Soybean ecotype. All the data points are shown on the plots. Data in A–F are presented as mean ± SD (n ≥ 4). Data in (A), (C), (D), and (F) were analyzed by *Student t*-test (two-side), asterisks indicate significant differences (**P* < 0.05). Data in (B) and (E) were analyzed by one-way ANOVA followed by Fisher’s LSD test (two-sided), asterisks indicate significant differences (**P* < 0.05; ***P* < 0.01).


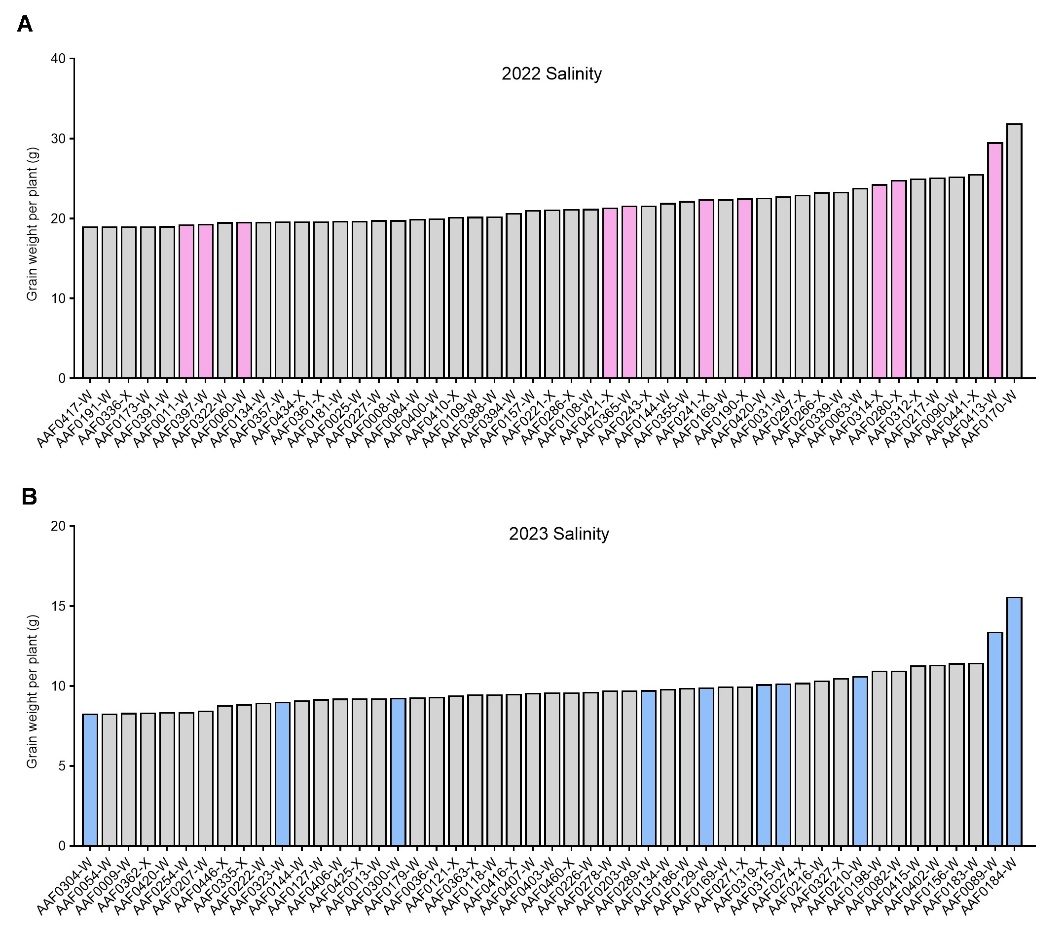


**Figure S18.** Superior yield performance of accessions carrying elite haplotype combinations under salinity stress. A–B) Ranking of soybean cultivars based on grain weight under saline conditions in 2022 (A) and 2023 (B). Cultivars carrying the elite combined haplotypes of *GmSIN1*, *GmRNF1a*, and *GmCSN5a* consistently ranked within the top 50. In each panel, carriers are highlighted in color: lavender in (A) and sky blue in (B). Non‑carriers are shown in gray in both panels.


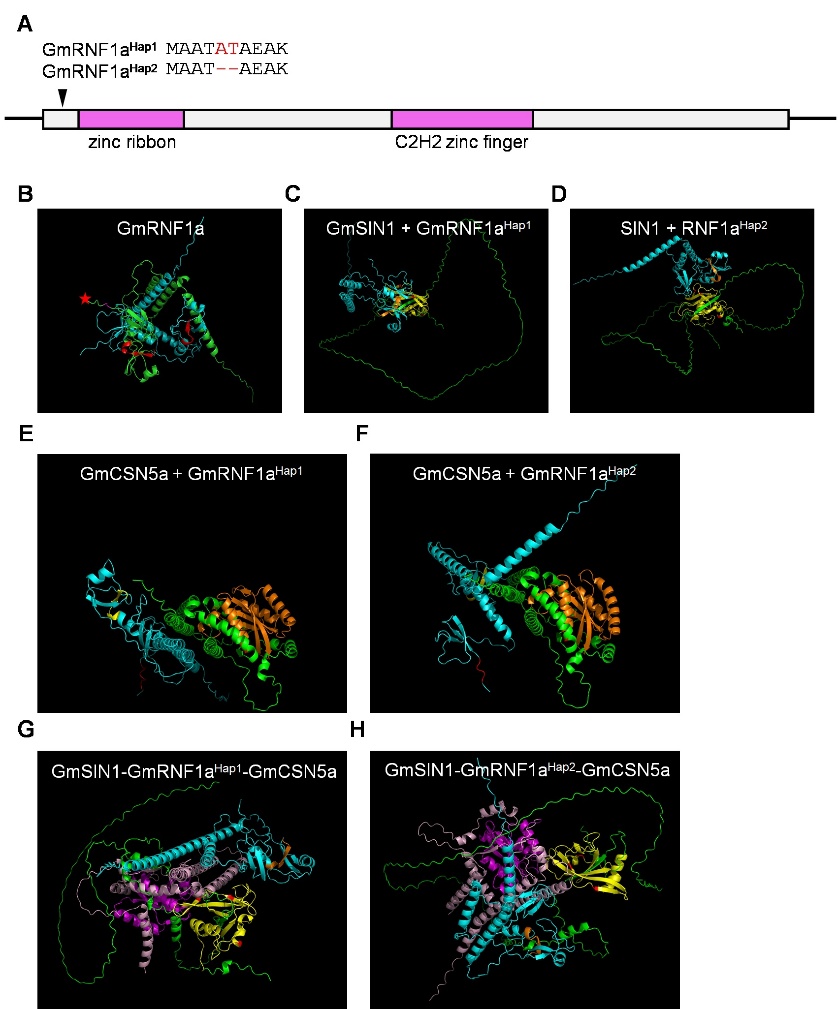


**Figure S19.** Structural basis for haplotype-dependent regulation within the GmSIN1-GmRNF1a-GmCSN5a module. A) Schematic of the GmRNF1a protein. Functional domains are colored lavender; haplotype-associated mutation sites are indicated by black triangles. B) Superimposed structures of the two GmRNF1a haplotype variants (GmRNF1aHap1, green; GmRNF1a^Hap2^, cyan). Mutation sites are marked with stars (★); the catalytic center is highlighted in red. C–D) Composite models of the GmSIN1 protein in complex with GmRNF1a^Hap1^ (C) or GmRNF1a^Hap2^ (D). The NAM domain of GmSIN1 is shown in yellow, its ubiquitination sites in red, and the catalytic center of GmRNF1a in orange. E–F) Conformational states of binary complexes formed between GmRNF1a haplotypes and GmCSN5a haplotypes. The catalytic center of GmRNF1a is colored yellow; the MPN domain of GmCSN5a is orange. G–H) Ternary complex structures resulting from interactions among GmSIN1, GmRNF1a haplotypes, and GmCSN5a haplotypes. Coloring is consistent: GmSIN1 NAM domain (yellow), ubiquitination sites (red), GmCSN5a MPN domain (magenta), and GmRNF1a catalytic center (orange).
